# Molecular evolution of *Phytocyanin* gene and analysis of expression at different coloring periods in apple (*Malus domestica*)

**DOI:** 10.1186/s12870-024-05069-6

**Published:** 2024-05-08

**Authors:** Miao Shao, Yongqing Feng, Shangwen Yang, Tong Feng, Fanwei Zeng, Shixiong Lu, Zonghuan Ma, Baihong Chen, Juan Mao

**Affiliations:** College of Horticulture, Gansu Agricultural University, Lanzhou, 730070 PR China

**Keywords:** Apple, Phytocyanin (PC), Identification, Molecular evolution, Expression analysis, Coloring period

## Abstract

**Background:**

PC (phytocyanin) is a class of copper-containing electron transfer proteins closely related to plant photosynthesis, abiotic stress responses growth and development in plants, and regulation of the expression of some flavonoids and phenylpropanoids, etc., however, compared with other plants, the PC gene family has not been systematically characterized in apple.

**Results:**

A total of 59 *MdPC* gene members unevenly distributed across 12 chromosomes were identified at the genome-wide level. The proteins of the MdPC family were classified into four subfamilies based on differences in copper binding sites and glycosylation sites: Apple Early nodulin-like proteins (MdENODLs), Apple Uclacyanin-like proteins (MdUCLs), Apple Stellacyanin-like proteins (MdSCLs), and Apple Plantacyanin-like proteins (MdPLCLs). Some MdPC members with similar gene structures and conserved motifs belong to the same group or subfamily. The internal collinearity analysis revealed 14 collinearity gene pairs among members of the apple *MdPC* gene. Interspecific collinearity analysis showed that apple had 31 and 35 homologous gene pairs with strawberry and grape, respectively. Selection pressure analysis indicated that the *MdPC* gene was under purifying selection. Prediction of protein interactions showed that MdPC family members interacted strongly with the Nad3 protein. GO annotation results indicated that the *MdPC* gene also regulated the biosynthesis of phenylpropanoids. Chip data analysis showed that (*MdSCL3, MdSCL7* and *MdENODL27*) were highly expressed in mature fruits and peels. Many cis-regulatory elements related to light response, phytohormones, abiotic stresses and flavonoid biosynthetic genes regulation were identified 2000 bp upstream of the promoter of the *MdPC* gene, and qRT-PCR results showed that gene members in Group IV (*MdSCL1/3*, *MdENODL27*) were up-regulated at all five stages of apple coloring, but the highest expression was observed at the DAF13 (day after fruit bag removal) stage. The gene members in Group II (*MdUCL9*, *MdPLCL3*) showed down-regulated or lower expression in the first four stages of apple coloring but up-regulated and highest expression in the DAF 21 stage.

**Conclusion:**

Herein, one objective of these findings is to provide valuable information for understanding the structure, molecular evolution, and expression pattern of the *MdPC* gene, another major objective in this study was designed to lay the groundwork for further research on the molecular mechanism of *PC* gene regulation of apple fruit coloration.

**Supplementary Information:**

The online version contains supplementary material available at 10.1186/s12870-024-05069-6.

## Background

Phytocyanins (PCs) are plant-specific type I blue copper protein (BCP) [1]. Structurally, PC proteins possess disulfide bonds and an open β-sandwich consisting of seven β-strands, which ensures the stabilization of PC proteins [2]. Previous studies have shown that PC proteins generally contain four structural domains [3, 4]: structural domain I is a Signal peptide (SP) located at the N-terminus that targets the protein to the endoplasmic reticulum; structural domain II is a Plastocyanin-like domain (PCLD) containing two cysteines (Cys) in the sequence; structural domain III is an Arabinogalactan protein-like region (ALR) glycoprotein structure that is similar to the structure of plant cell walls and can integrate proteins into plant cell walls; structural domain IV is characterized by a glycosylphosphatidylinositol anchor signal (GAS), which enabled target proteins to anchored to the cell membrane [5]. Different structural domains result in PC proteins exercising different biological functions, however, this PCLD structural domain is indispensable for PC proteins [4].

Uclacyanin-like proteins (UCLs), Stellacyanin-like proteins (SCLs), Plantacyanin-like proteins (PLCLs), and Early nodulin-like protein (ENODLs) [6, 7] are major members of the PC proteins. Except for the ENODLs family members, all other members of the PCs have intact copper ion binding sites [8]. Among them, the copper ligands of both UCLs members and PLCLs members are composed of two His, one Cys and one Met, but UCLs are chimeric glycoproteins, while PLCLs are non-glycoproteins without such glycoprotein-like structural domains [9]. Unlike UCLs and PLCLs copper ligands, Gln is replaced by Met in the residues of SCLs copper ligands. It is worth noting that in addition to the N-glycosylation site by asparagine (Asn) residues linking SCLs and UCLs, there are also O-glycosylation sites via serine (Ser) and hydroxyproline (Hyp) residues [9]. Several previous studies have shown that because an arabinogalactan protein-like region is present in most Arabidopsis and rice PCs, they are also classified as a subfamily of the AGP superfamily (ALR) [8, 10].

Extensive studies have shown that in plants PCs were involved in a variety of biological processes including; plant cell differentiation, reproductive processes, somatic embryogenesis and stress response [11–13]. In *Arabidopsis thaliana*, PCs were involved in reproductive processes, and overexpression of the *AtPC* gene inhibited pollen grain germination [11]. Phytocyanin proteins isolated from lily stigmas also induced pollen tube chemotaxis [14]. Similarly, overexpression of *OsUCL8* inhibited the normal pollination of rice and thus affected the fruiting rate of rice, while knockdown of *OsUCL8* and overexpression of miR408 significantly increased the pollen germination rate of rice [15]. The ENODL protein encoded by At3g20570 in sieve tube molecules in Arabidopsis participated in and regulated the reproductive growth in Arabidopsis [12]. In addition, some members of the PCs are redox components of the cell wall, for example, a *PC* gene associated with the formation of lignin was identified in pods [16], and *PC* genes were likewise involved in the process of xylem differentiation in torch pine [17]. There were many studies on PC has participated in biotic and abiotic stress response. ENODL proteins have been extensively studied in the process of nodulation in legumes, and *GmENODL55* in soybean was expressed only in soybean rhizoma cells after infestation with a slow-growing type of soybean rhizobacteria [18]. In peas, *VsENOD5* was expressed in rhizobial cells after infestation with *Rhizobium spp* [19]. As well as *MtENODL27/28* as essential factors for *Medicago truncatula* rhizobium infestation and rhizoma development, inhibition of *MtENODL27* and *MtENODL28* expression impeded rhizobium infestation and rhizoma formation, while *MtENODL27* and *MtENODL28* expression was up-regulated in rhizobium-infested root cells [4]. Previous studies have shown that *PC* genes are also involved in the process of plant response to abiotic stresses. In poplar, most *PtPC* gene expression level was up-regulated under the treatment conditions of salt stress and drought stress [20]. Similarly, *PC* genes in *Boea crassifolia* and maize responded to drought stress and salt stress [21, 22], Overexpressed the *BcBCP1* gene enhanced drought stress resistance in transgenic tobacco [21]. *NtENODL* gene expression was down-regulated in most tobacco under low temperatures and MeJA-induced conditions [23]. Earlier studies confirmed that the *AtBCB* gene (An Arabidopsis blue copper-binding protein gene) inhibited aluminum uptake and protected plants from aluminum toxicity [24, 25].

Anthocyanidins as a flavonoid secondary metabolite, regulate the color of various tissues and organs in plants [26]. Similarly, anthocyanins are also involved in abiotic stress responses in plants and can protect plants from UV radiation damage [27]. Therefore, the production of anthocyanins is considered as an adaptive response of plants under adverse growth conditions [27–29]. Previous studies have found that many transcription factors and structural genes regulate the process of anthocyanin biosynthesis [26, 30]. An interesting result of the previous study was silencing of the *GhENODL6* gene in cotton led to a dramatic decline in both the phenylalanine ammonia-lyase (*PAL*) gene expression levels and 4-coumarate-coenzyme a ligase (*4CL*) gene expression levels in the phenylalanine ammonia-lyase pathway [31]. Extensive studies have indicated that *PAL* genes and *4CL* genes participate in anthocyanin synthesis in plants [32, 33]. In rice, an interesting finding was that OsmiR528 directly targeted OsUCL23 thereby regulating flavonoid metabolism levels [34]. Prior research has proved that flavonoid metabolites can influence plant coloration [35]. However, compared to the previous research on *PC* genes in response to reproductive processes and stress response in plants, there is still a lack of work on whether *PC* genes regulate plant colorization. It is an interesting issue whether *PC* genes directly or indirectly regulate the expression of genes related to anthocyanin synthesis, which in turn are involved in fruit coloration.

Genome-wide systematic analysis can provide an effective way to identify gene family members and elucidate their biological roles. However, the *PC* genes have not yet been characterized in the whole apple genome. Previous studies have focused on *PC* genes regulating plant growth and development and coping with abiotic stresses. Given that *PC* genes can regulate the production of some flavonoids related to plant coloration and some genes of the anthocyanin synthesis pathway, it is interesting to see whether *PC* genes can also, directly and indirectly, regulate fruit coloration. Using bioinformatics methods aimed at identifying and characterizing members of the apple PC gene family members. Chromosomal localization, gene structure, conserved motifs, protein secondary and three-dimensional structures, gene collinearity, selection pressure, codon preference and relative synonymous codon usage, predicted protein interaction pathways, gene ontology annotation, cis-acting elements and plant organs chip data were analyzed at *MdPC* gene members. The *MdPC* gene expression level was analyzed by qRT-PCR for different coloring periods of apples after de-bagging, which will provide insight to reveal the biological functions of MdPC family members.

## Results

### *MdPC* gene phylogenetic relationships analysis

**Fig. 1:**
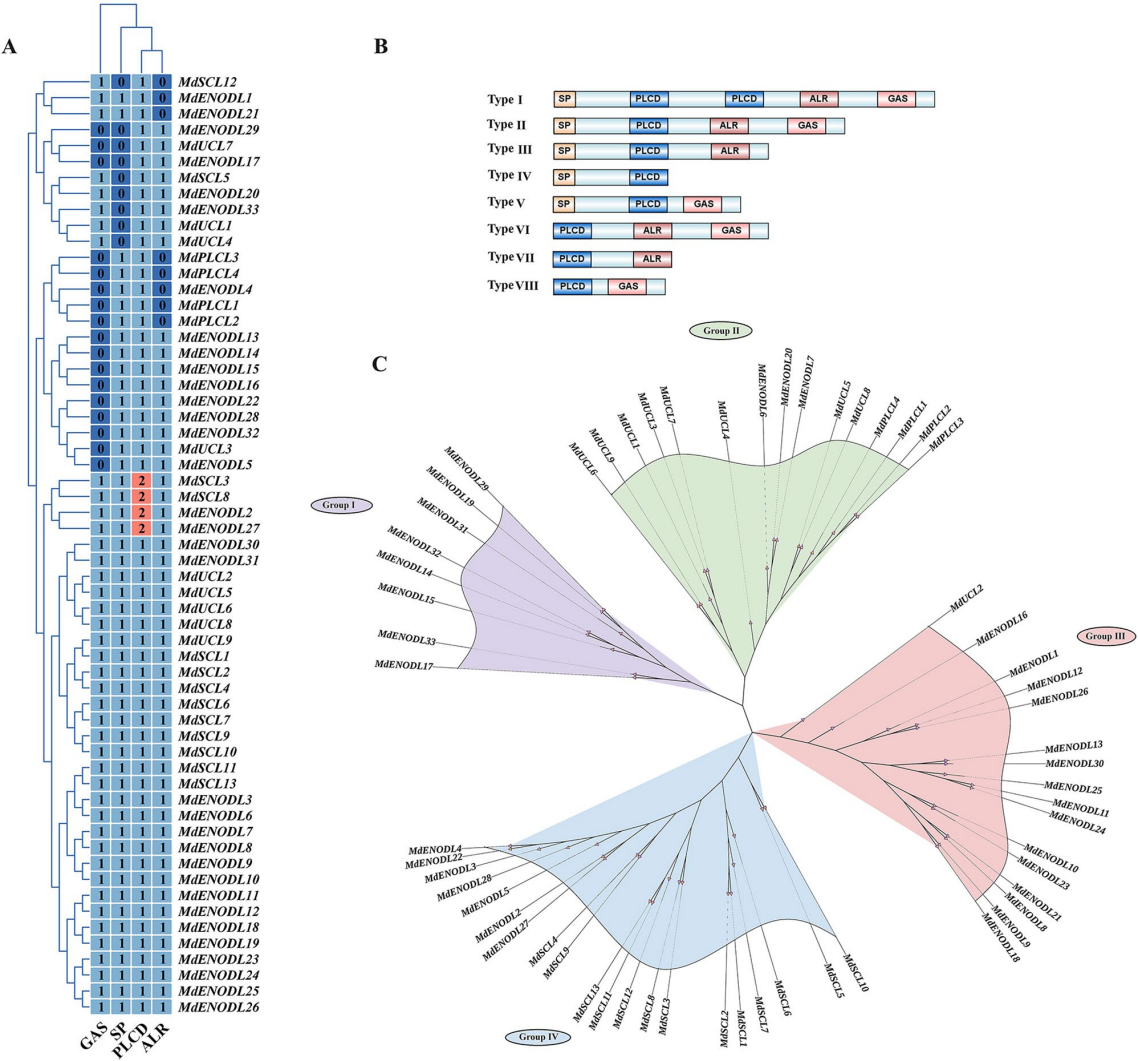
Phylogenetic analysis of MdPC gene family in apple. **A** Four types of structural domains of the MdPC members Plastocyanin-like domain (PCLD), Arabinogalactan protein-like region (ALR), Signal peptide (SP), Glycosylphosphatidylinositol anchor signal (GAS). **B** Classification of PC members into different types based on different structural domains **C** Phylogenetic analysis of MdPC proteins. Apple Early nodulin-like proteins (MdENODLs), Apple Uclacyanin-like proteins (MdUCLs), Apple Stellacyanin-like proteins (MdSCLs), and Apple Plantacyanin-like proteins (MdPLCLs). The Groups were marked by a colorful background

Further classification of MdPC protein members using four functional domains (SP, ALR, PLCD and GAS) (Fig. 1A), and based on the composition of the four structural domains in MdPC proteins, MdPC proteins can be classified into eight types (I-VIII) (Fig. 1B). A total of 50 MdPC proteins had N-terminal signal peptides, except for members of types VI, VII and VIII. The members of types IV, V and VIII did not contain ALR (Arabinogalactan protein-like region), and four MdPC proteins (MdSCL3/8, MdENODL2/27) belonging to type I members had two PLCD domains, and it was hypothesized that the duplication of the PLCD structural domain may have originated from the duplication of the PLCD structural domain region during the evolution of the apple PC sequence. In addition, 42 MdPCs belonging to type I, II, V, VI, and VII have GAS, implying that these proteins may be localized to the cell membrane. Among these MdPC members, members of gene families I, II and III with both SP and ALR functional domains can be considered as chimeric AGPs.

Some MdPC members with similar functional domain compositions were more inclined to cluster together on the phylogenetic tree, so a phylogenetic tree was constructed using multiple sequence alignment to further analyze the MdPC members (Fig. 1C). The 59 members of the phytocyanin gene contained a conversed phytocyanin protein domain, and the results demonstrated that phytocyanin was highly conserved in the apple. Based on the differences in copper-binding sites as well as glycosylation sites, MdPC proteins can be classified into four subfamilies: four members are attributed to plantacyanin-like proteins (MdPLCLs) nine members to uclacyanin-like proteins (MdUCLs), thirteen members to stellacyanin-like proteins (MdSCLs), and the remaining 33 MdPCs that do not contain a copper-binding site are classified into the subfamily of early nodulin-like proteins (MdENODLs). These sequences are named MdSCL1 to MdSCL9, MdPLCL1 to MdPLCL4, MdENODL1 to MdENODL33, and MdUCL1 to MdUCL9, respectively. According to the genetic distance, the phytocyanin gene family was divided into four groups, named Group I, II, III, IV. Among them, 8 MdPCs belonging to early nodulin-like proteins (MdENODLs) were categorized into group I. Group II contains all members of the plantacyanin-like proteins (MdPLCLs), and these MdPLCLs members are of type IV, Group III contains one member of the MdUCLs in addition to 15 members of the MdENODLs, and notably, Group IV contains all members of the MdSCLs in addition to six members of the MdENODLs. And most of the MdSCLs and MdUCLs belong to type II.

### Analysis of *MdPC* physicochemical properties and chromosomal localization

**Fig. 2:**
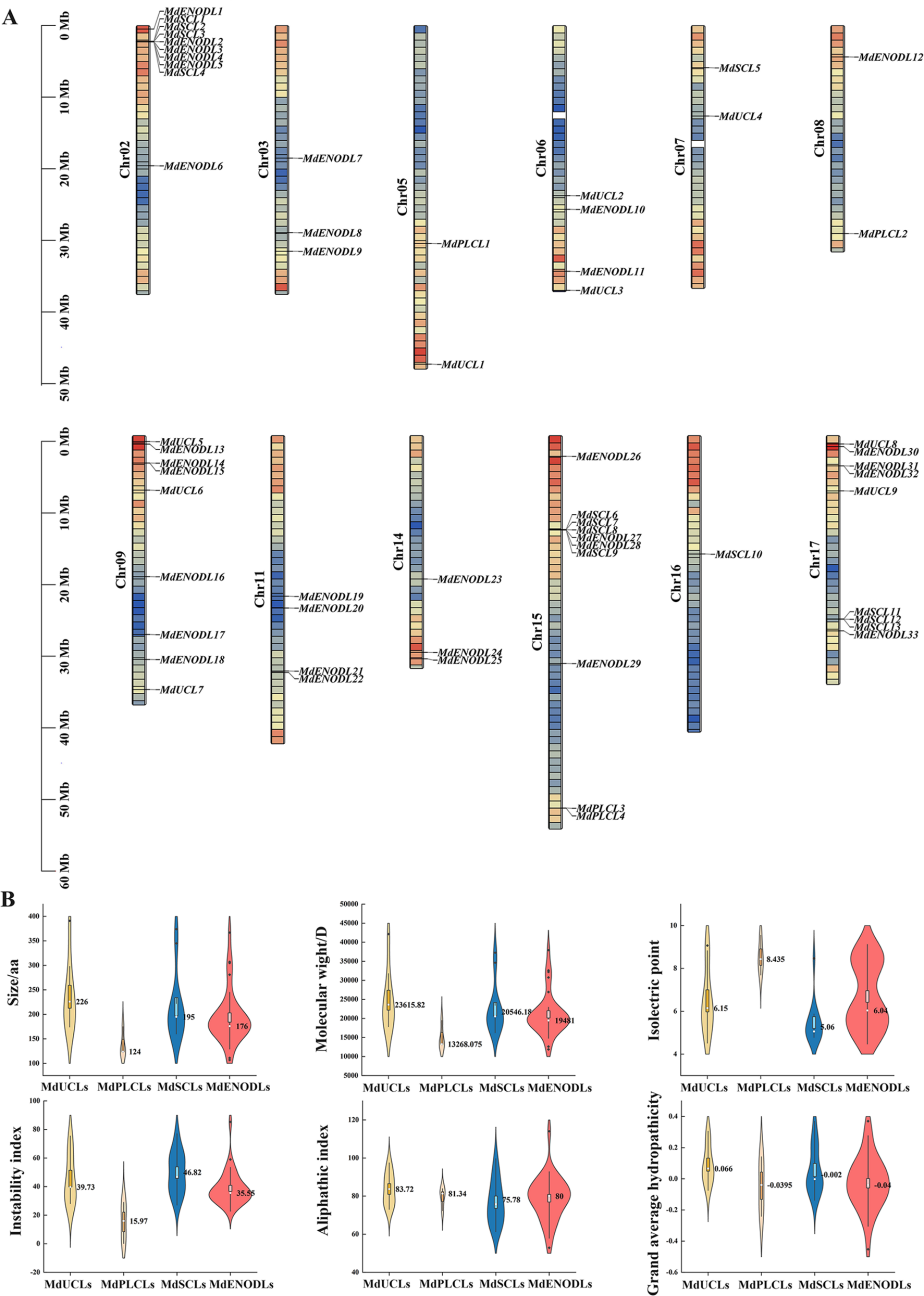
Distribution of *MdPC* genes on different apple chromosomes and MdPC protein physicochemical property Violin Diagram. **A** Chromosomes are indicated by colored bars. The left scale indicates the chromosome length (Mb). The position of the *MdPC* gene is marked with a black line. The different colors represent gene density, where red indicates high-density regions and blue indicates low-density regions. **B** Violin box plots physicochemical properties for MdPC proteins. Black dots and white dots represent outliers and averages, respresently

In this study, 59 candidates for *MdPC* genes were identified (Supplementary Table S1). 59 *MdPCs* were renamed consecutively according to apple chromosome positions 2nd − 17th (Fig. 2A). The 59 *MdPC* genes were unevenly distributed on twelve chromosomes. Chromosomes 2 and 15 contain the highest number of *MdPC* genes with 10 *MdPC* genes each, followed by chromosomes 9 and 17, both with 9 *MdPC* genes. chromosome 16 has the lowest number of *MdPC* genes with only one. It indicates that there is no significant correlation between the number of *MdPC* genes on each chromosome and chromosome length in apple. There were many gene clusters, such as chromosome 2 with 10 *MdPC* genes, and chromosome 17 with 9 *MdPC* genes. The presence of these gene clusters indicates that the *MdPC* gene is replicated in tandem, and thus leads to an increase in the number of *MdPC* gene members.

MdPLCLs had lower average Amino acid sizes, Molecular weight, Aliphatic index, Instability index and Grand average of hydropathicity than the other three subfamily members, except for the average Isoelectric point, which was higher than the other three subfamily members (Fig. 2B). The CDS length of 59 *MdPC* genes varied from 324 to 1176, encoding polypeptides of 108 to 392 amino acids, with a predicted molecular mass range from 11.87 to 42.15 kD. The theoretical pI (isoelectric point) ranged from 4.47 (MdENODL22) to 9.55 (MdPLCL4) and included 20 basic proteins (pI > 7.5), 2 neutral proteins (pI 6.5–7.5) and 37 acidic proteins (pI < 6.5). The aliphatic index (AI) of the MdPC protein ranged from 52.89 (MdENODL9) to 114.05 (MdENODL17), and the Instability index (II) ranged from 0.13 (MdPLCL3) to 85.3 (MdENODL9) (Supplementary Table S1). According to the Grand average of hydropathicity (GRAVY), The hydropathicity to hydrophilicity protein ratio was 1.03 and included 31 hydropathicity and 28 hydrophilicity proteins. It indicates that most MdPC proteins in apple were hydropathicity proteins.

### MdPC secondary structure and subcellular location prediction analysis

**Fig. 3:**
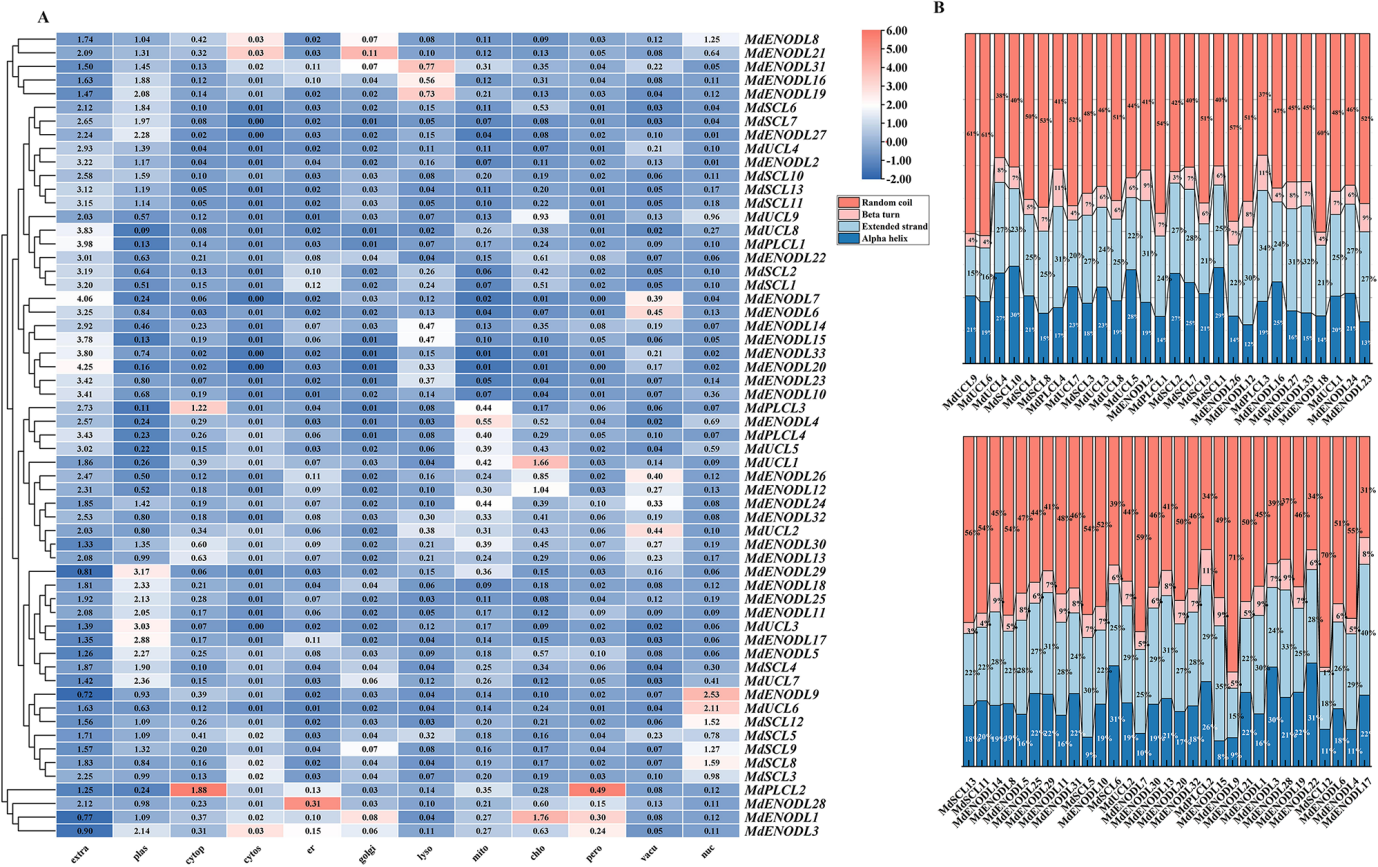
The secondary structure and subcellular location prediction of MdPC proteins. **A** Subcellular location prediction of MdPC proteins. Extracellular (extra) and plasma membrane (plas), cytoplasmic (cytop), cytoskeleton (cytos), endoplasmic reticulum (er), lysosomes (lyso), mitochondrial (mito), chloroplast (chlo), peroxisome (pero), vacuole (vacu) and nuclear (nuc). Red for high expression, blue for low expression **B** The secondary structure MdPC proteins. The secondary structure consists mainly of Random coil, Beta turn, Extended strand and Alpha helix. Different colors represent different secondary structures

The *MdPC* genes subcellular localization prediction analysis showed (Fig. 3A) that the apple phytocyanin genes were mainly located in the extracellular and plasma membrane. This result may be associated mostly with genes having GAS. It is noteworthy that *MdPLCL2* is mainly located in the cytoplasm. The remaining *MdPC* genes were predicted to be located in the plasma membrane, cytoplasmic, cytoskeleton, golgi, chloroplast, peroxisome, mitochondrial and vacuole areas. Apple phytocyanin protein secondary structure (Fig. 3B) showed that the secondary structure of the MdPC family members consisted mainly of Random coils. The proportions of Alpha helix and Extended strand distributions were 7.75–31.33% and 14.99–39.64%, in that order. The beta turn was mainly distributed between 1.25%∼11.29%, and the Random coil was mainly distributed between 30.63%∼71.39%.

### Three-dimensional structure and structural domain analysis of apple PC protein

**Fig. 4:**
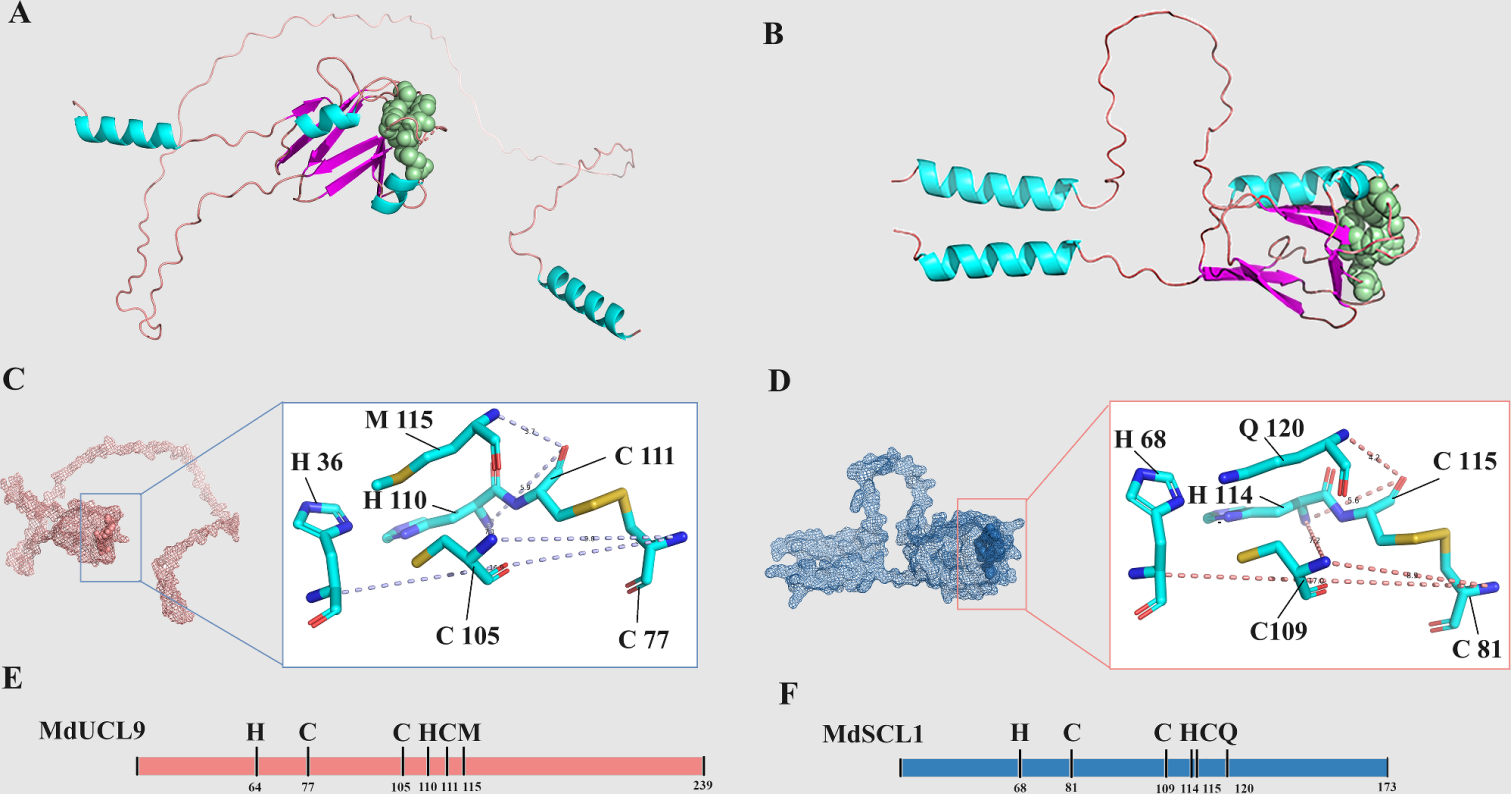
Three-dimensional structure analysis of MdPC proteins. **A**, **B** Comparison of three-dimensional protein structures of MdUCL9 and MdSCL8. The different secondary structures are represented by different colors, with the spheres representing the conserved amino acid residues of MdPC with bound copper ions. **C**, **D** Analysis of conserved amino acid residues binding copper ions. **E**, **F** Amino acids sequences representing MdUCL9 and MdSCL8. His, Cys, Gln and Met (H, C, Q and M), Numbers represent amino acids locations

For an insight study of the structure of MdPC proteins, a protein structure prediction was performed using SWISS-MODEL software. The three-dimensional structures of MdUCL9 and MdSCL3 proteins were mapped based on the composition of the protein’s secondary structures. (Fig. 4A-B). The amino acids represented by these protein sequences were also labeled according to the conserved amino acid residues that can bind copper ions and the cysteine residues involved in disulfide bond formation. For example, the conserved sequence H-C-C-H-M of MdUCL9, and H-C-C-C-H-Q of MdSCL3 (Fig. 4C-F). The three-dimensional structure prediction further validates the accuracy of the conserved motif prediction for the MdPC proteins.

### *MdPC* gene structure and motif composition analysis

**Fig. 5:**
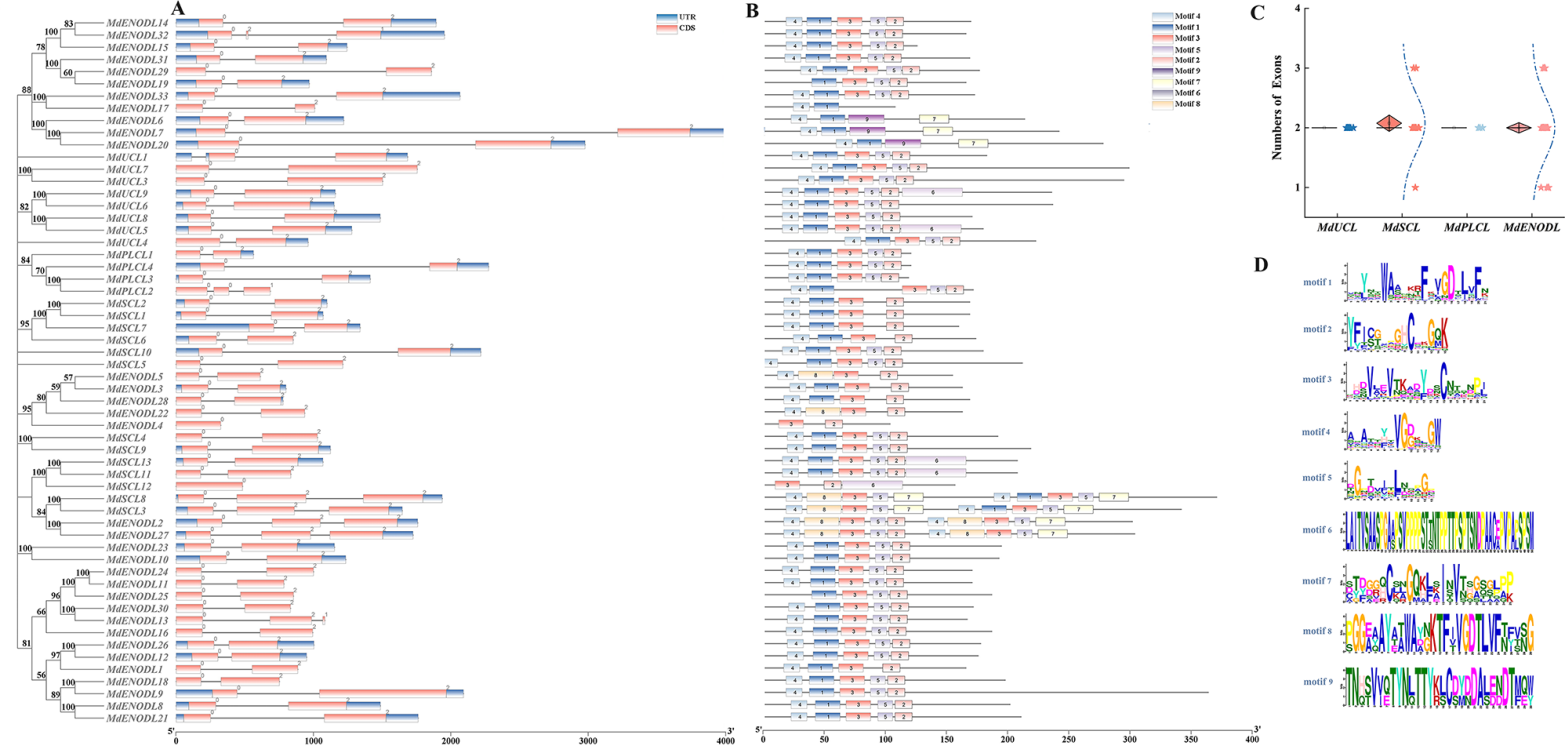
*MdPC* gene structure and motif analysis. **A** MdPC conserved motifs, different motifs are marked with different colors and the numbers above represent different motifs. **B** The exon-intron structure of the *MdPC* gene, numbers represent the number of splice sites. **C** Box plot of the number of CDS of *MdPC* genes. Boxes are represented by diamond-shaped squares, points are represented by stars, and the blue line shows the trend in the distribution of CDS numbers. **D** Specific amino acids for different motifs

To identify structural features in the 59 MdPC proteins, the number of conservative patterns predicted using the MEME software is 9 (Fig. 5B and D). Motif 1, 2, 3 and 4 existed in most MdPC. However, the motif 7, 8 and 9 only existed in several MdPCs. It was worth noting that MdSCL3, MdSCL8, MdENODL2 and MdENODL27 contain both two motif 3, two motif 4, and two motif 5. *MdENODL3* and *MdENODL27* genes contained longer introns in the graph. Among the gene structures analyzed, most members except *MdSCL12* and *MdENODL4* have only one CDS sequence (Fig. 5A). It was interesting to note that both MdUCL subfamily members and MdPLCL subfamily members have two exons, while MdSCL subfamily members and MdENODL subfamily members have the same distribution of exon numbers, which suggests that they have a similar function during the evolutionary process (Fig. 5C). Since, the motifs and gene structure of *MdSCL3* and *MdSCL8* genes were structurally similar, it speculated that *MdSCL3* and *MdSCL8* may perform similar functions.

### The *MdPC* gene collinearity analysis

**Fig. 6:**
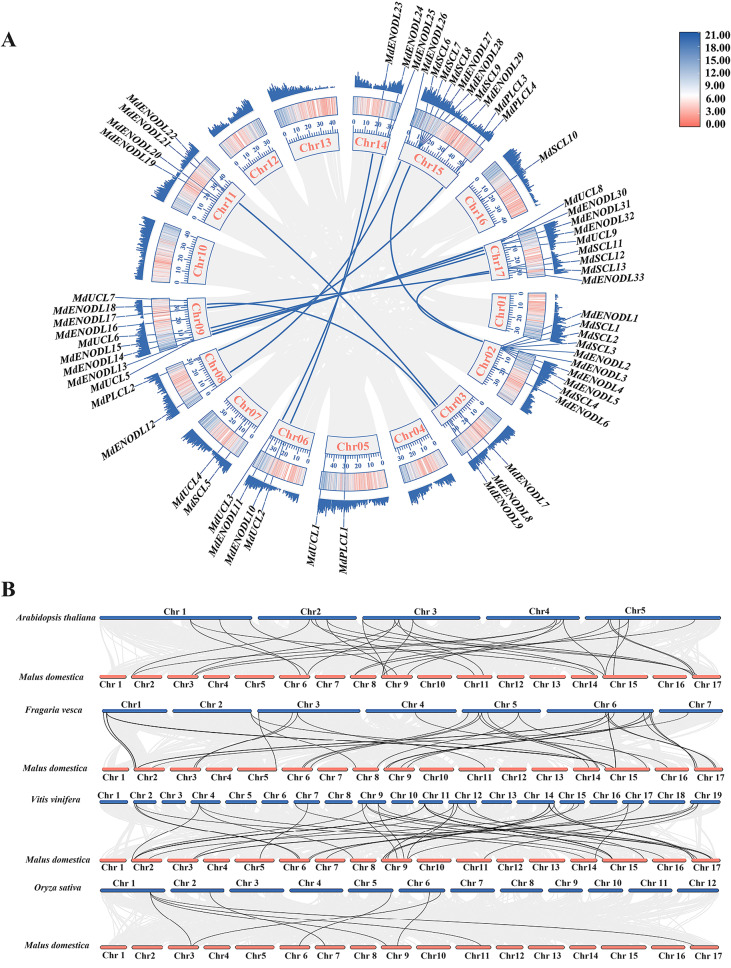
Collinearity relationship analysis of *MdPC* gene. **A** Collinearity relationship of *MdPC* family genes in apple. In the circles, collinearity gene pairs between *MdPC* genes are represented by blue curves. The outermost and second outermost circles are the two expression types of gene density. The gray line in the background indicates that the apple genome has collinearity gene pairs The Red color has a higher density and the blue color has the lowest density. **B** Collinearity relationships of *MdPC* genes between apples and four representative plant species. The black line highlights the collinearity of *PC* gene pairs

The collinearity analysis revealed that there were fourteen pairs of collinear- relationships gene pairs were found in apple, such as *MdSCL3/MdSCL8, MdENODL4/MdENODL28, MdUCL5/MdUCL8* and *MdENODL26/MdUCL9* (Fig. 6A). Thus, the *MdPC* gene may have amplified family members through gene duplication during evolution. To further illuminate the phylogenetic mechanism and homology of the *MdPC* gene, interspecies collinear-relationship analysis maps were built for apple and four representative species such as *Arabidopsis thaliana*, rice (*Oryza sativa* L.), strawberry (*Fragaria vesca*), and grape (*Vitis vinifera* L.) (Fig. 6B). Among them, the results show that apple/Arabidopsis, apple/grape, apple/strawberry, apple/rice had 29, 35, 31 and 8 homologous pairs of genes, respectively. Overall, this indicates that the *MdPC* gene has evolved to be more distantly related to monocotyledon rice and more closely related to dicotyledon grape.

### *MdPC* gene evolution selection pressure and codon usage bias analysis

**Fig. 7:**
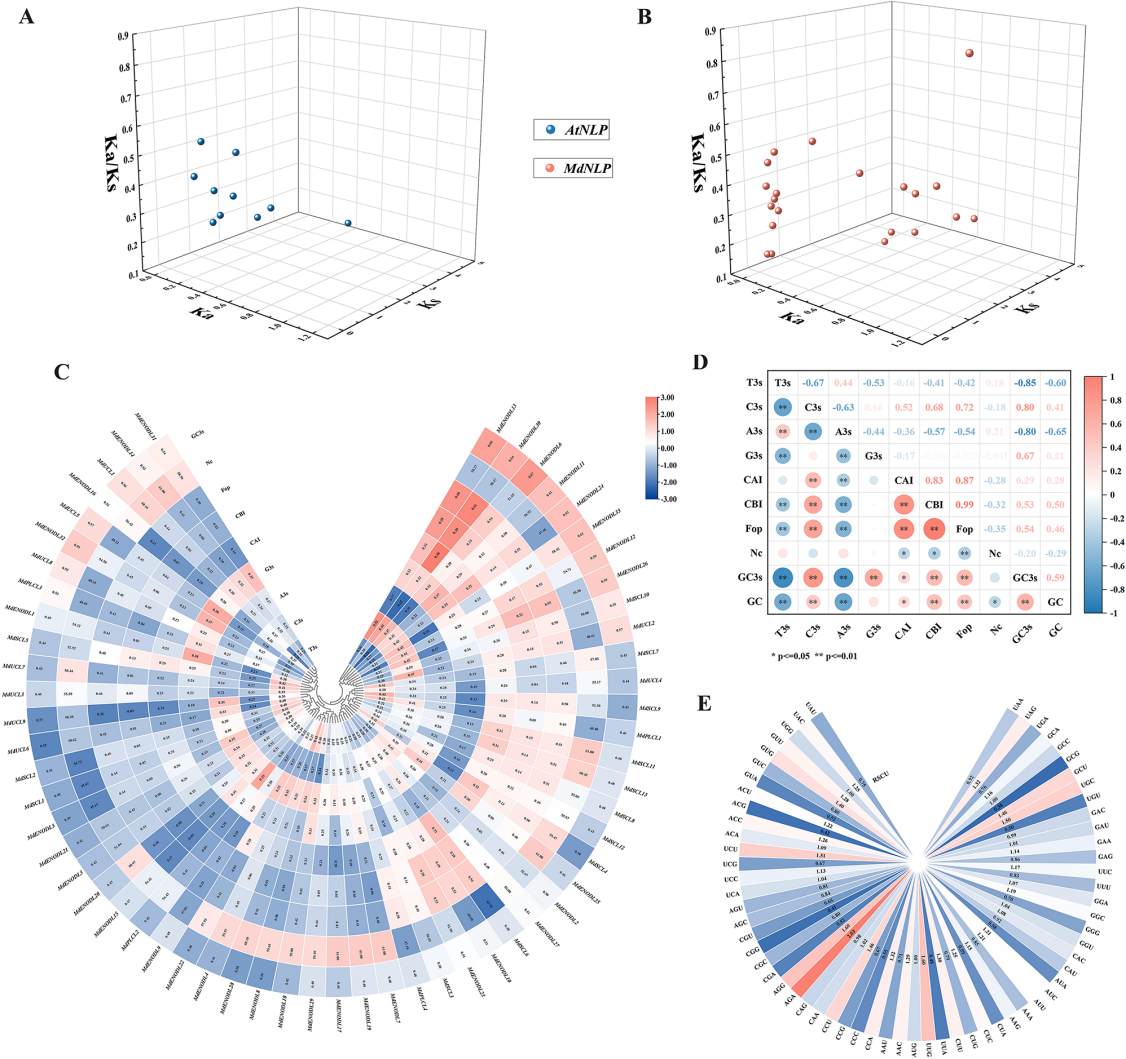
*MdPC* gene evolutionary selection pressure and codon usage bias analysis. **A** Ka/Ks analysis of *PC* collinearity relationship gene pairs in Arabidopsis. **B** Ka/Ks analysis of collinearity relationship *PC* gene pairs in apple. **C** Synonymous codon preference and correlation analysis of *MdPC* gene. the U, C, A and T of the codon third site (U3s, C3s, A3s, G3s and T3s), codon adaptation index (CAI), codon preference index (CBI), frequency of optimal codon usage (FOP). effective codon number (NC), GC content of the third position of synonymous codon (GC3s). **D** Correlation analysis of *MdPC* gene codon. Red indicates a positive correlation, Blue indicates a negative correlation, and white indicates no correlation. **E** Relative synonymous codon usage (RSCU) of MdPC.

To explore PC protein evolutionary relationships, both apple and Arabidopsis sequences were used to calculate evolution selection pressure. A large number of Ka/Ks are derived from gene pairs with collinear relationships in the PC gene family. In this study, the whole non-synonymous mutation frequency (Ka) was less than 0.868, but silent mutation (Ks) was more than 0.243 (Fig. 7A). In addition, the Ka/Ks value of the *MdPC* and *ATPC* gene members were less than 1(Fig. 7B). Therefore, an interesting conclusion was harvested, they might have a purification selection. This suggests that *PC* genes are relatively conserved during evolution in different species.

The NC values for the MdPC gene family ranged from (*MdSCL6*) 43.16-61.00 (*MdSCL11*) (Fig. 7C). The CAI and CBI values for *MdENODL30* were the highest. The GC range for the 59 *MdPC g*enes was (0.41–0.58), indicating that the apple *MdPC* genes were not inclined to GC. *MdPC* genes with G/C3s values greater than 0.5 accounted for 54.23% of the total, indicating that *MdPC* genes prefer termination codon with A/T. Notably, the GC3s of *MdPC* genes were significantly positively correlated with G3s and C3s and negatively correlated with T3s and A3s (Fig. 7D). More importantly, the FOP of the *MdPC* gene was significantly positively correlated with CBI and CAI. According to RSCU figure, it became apparent that 18 codons (UUU, UUG, AUU, GUU, GUG, UCA, CCA, ACU, GCU, GCA, UAU, CAU, CAG, AAU, AAG, GAA, GAG, AGA) were used most frequently in the MdPC gene family, and that most codons had RSCU > 1 (Fig. 7E). These codons can be used as *MdPC* optimal codons. The *MdPC* gene has no preference for the valine encoded by UGG and the methionine encoded by AUG.

### MdPC gene family cis-acting elements analysis

**Fig. 8:**
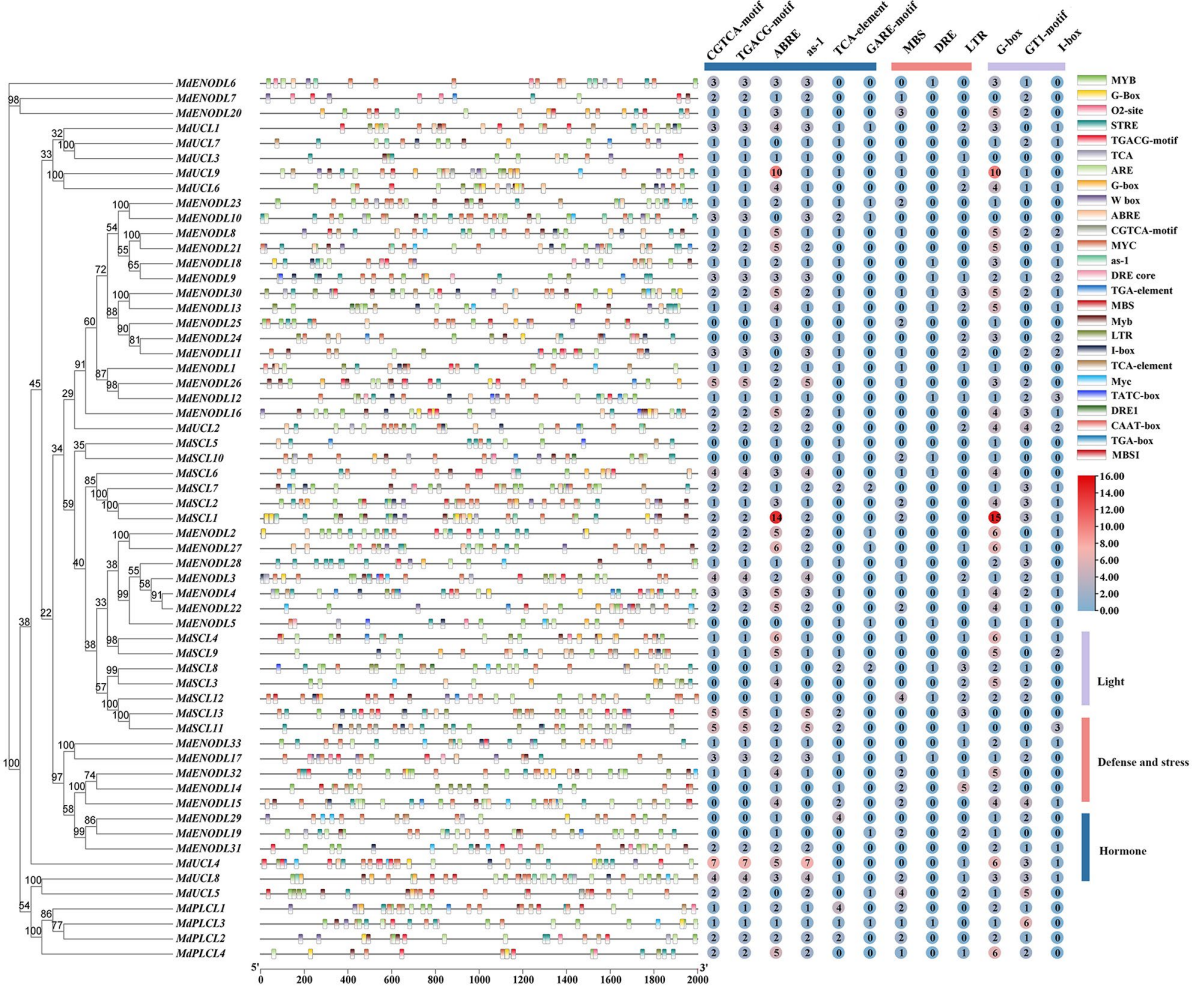
The first 2000 bp of cis-acting elements of 59 *MdPC* genes. Different elements are labeled with different colors. Purple represents light-responsive elements, red represents stress-defense elements, and blue represents hormone-acting elements, The number in the circle represents the number of elements

*MdPC* gene promoter regions involved different types of cis-acting regulatory elements, and cis-acting elements played an important role in the regulation of gene expression. To investigate the types of cis-acting elements and explore the mechanism of *MdPC* genes, 2.0 kb of upstream sequence of 59 *MdPC* genes were submitted to the PLACE online site. The results indicated that the MdPC gene family contained phytohormones reaction elements such as Methyl jasmonate (MeJA), Auxin (IAA), Salicylic acid (SA), Gibberellin (GA) and Abscisic acid (ABA) elements., light response elements, (I-box, G-box, GT1-motif) and many elements related to stress response elements, such as low-temperature response element (LTR), drought-induced MYB-binding site (MBS), and drought- and salt-stress-responsive element DRE (Fig. 8). The number of cis-acting regulatory elements was different among the *MdPC* genes, Through analysis of *MdPC* genes cis-acting elements, it found that 47 *MdPC* genes were related to MeJA, 50 *MdPC* genes were related to abiotic stress, only 11 *MdPC* genes were unrelated to GA cis-acting regulatory elements, four *MdPC* genes were unrelated to SA cis-acting regulatory elements, 53 *MdPC* genes were related to ABA cis-acting regulatory elements, otherwise, only three *MdPC* genes unrelated to light response elements. It was interesting to note that the MYB binding site involved in flavonoid biosynthetic genes regulation (MBSI element) in *MdSCL5*, *MdENODL3/7/29*. These results suggest that because a large number of *MdPC* genes have different types of cis-acting elements in their promoter regions different *MdPC* genes may perform different functions.

### Gene chip expression profile of the MdPC gene family in apple organs

According to the spatio-temporal expression characteristics of plant tissues and organs, 59 *MdPC* genes can be roughly classified into three types (Supplementary Fig. S1). The first type is that *MdPC* genes are expressed at low levels or not expressed in all tissues and organs. Examples include *MdSCL5*, *MdENODL1*, and *MdENODL4*. The second type is that *MdPC* genes are expressed in almost all tissue organs but have higher expression levels in individual tissue organs, such as *MdUCL1* was expressed most highly in receptacles and *MdSCL3* with the highest expression in ripe fruit peel. The third type of *MdPC* genes are highly tissue-organ specific and are highly expressed only in specific tissues, e.g., *MdENODL13*, and *MdENODL24* are most highly expressed only in flowers. *MdSCL9* and *MdUCL4* are most highly expressed only in whole seedling. It was interesting to find that *MdUCL2* was the highest expression *MdPC* gene found in the stigmas, styles, anthers and pollon. In petals, sepals, flowers and dormant buds *MdSCL7* expression was highest. It also found that *MdSCL7, MdSCL3* and *MdUCL1* were highly expressed in fully-developed leaves. It is noteworthy that most of the *MdUCL* subfamily members were highly expressed in apple whole seedlings, implying that the *MdUCL* subfamily members may play a crucial role in apple whole seedling development.

**Fig. 9:**
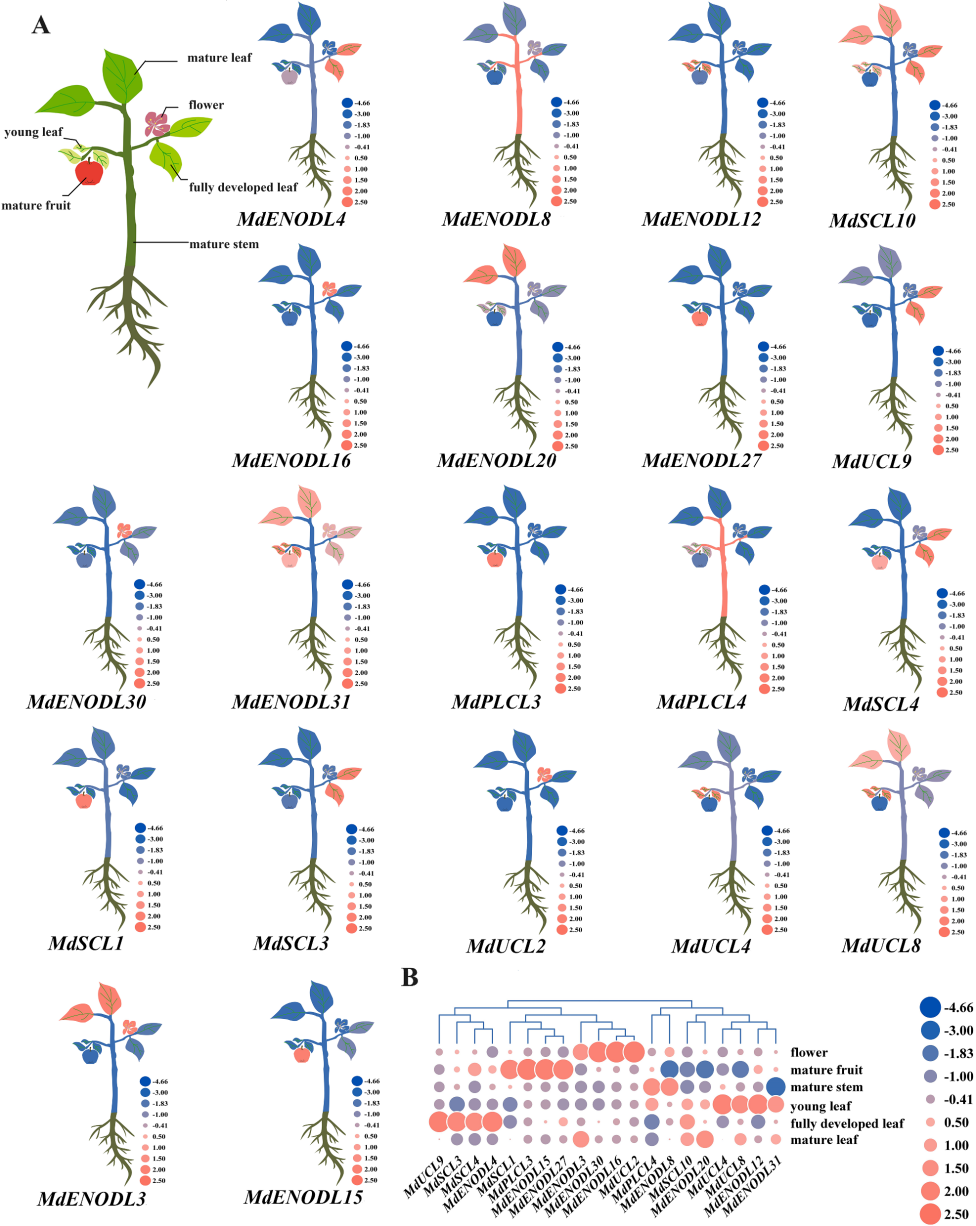
Heat map analysis of *MdPC* genes in different plant organs **A** Expression of the *MdPC* gene in different apple organs. **B** Heatmap of the expression of 20 *MdPC* genes. Red and blue represent up- or down-regulated expression levels, respectively

For further elucidation of the differences in the expression of different groups of MdPC members in plant organs, 20 *MdPC* genes were randomly selected from four groups and analyzed for their expression levels in six plant organs (Fig. 9), among which *MdENOD16/30* and *MdUCL2* from the III groups had a high degree of expression in flowers and *MdENODL15* and *MdENODL27* from I group and IV group in mature fruits, and *MdSCL10* was more highly expressed in the three types of leaves than the other genes. *MdSCL10* had a higher degree of expression in the three types of leaves compared to the other genes.

### Apple phytocyanin protein interactions prediction and *MdPC* gene annotation

**Fig. 10:**
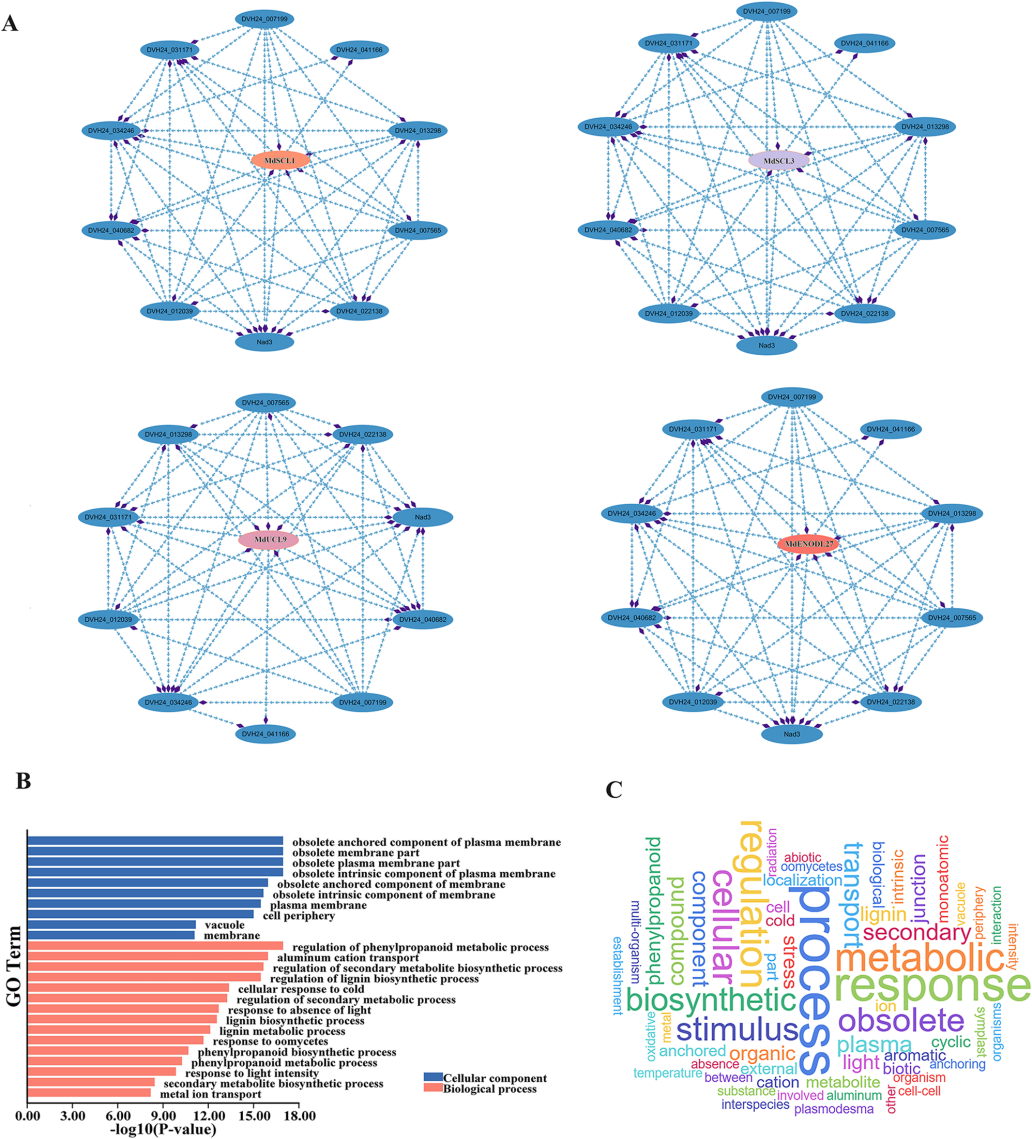
Protein interaction analysis and *MdPC* gene ontology annotation. **A** Predictive analysis of four MdPC proteins interacting with other proteins. **B***MdPC* gene ontology annotation. Red represents the biological processes, blue represents the cellular component. **C***MdPC* gene ontology annotation word clouds

Interaction prediction of four representative MdPC proteins with other proteins showed that four MdPC proteins (MdSCL1, MdSCL3, MdUCL9 and MdENODL27) interacted with other proteins. These protein interactions formed a protein interaction network (Fig. 10A). MdPC protein could interact with DVH24_03424 (Oxidored_q6 domain-containing protein. MdSCL1, MdSCL3, MdUCL9 and MdENODL27 can also interact with Nad3 proteins (Core subunit of mitochondrial membrane respiratory chain NADH dehydrogenase).

GO annotation of the 59 *MdPC* genes studied revealed their involvement in binding processes, catalytic activity, bioregulation, stimulus-response and abiotic stress response (Fig. 10B). The frequency of GO terms was visualized by the generated word cloud (Fig. 10C). These results also demonstrated the importance of *MdPC* in abiotic stress response (cold stress, response to light deficiency), and it is noteworthy that the *MdPC* gene also regulates the biosynthesis of phenylpropanoids.

### Expression analysis of *MdPC* gene members in different apple coloring periods

**Fig. 11:**
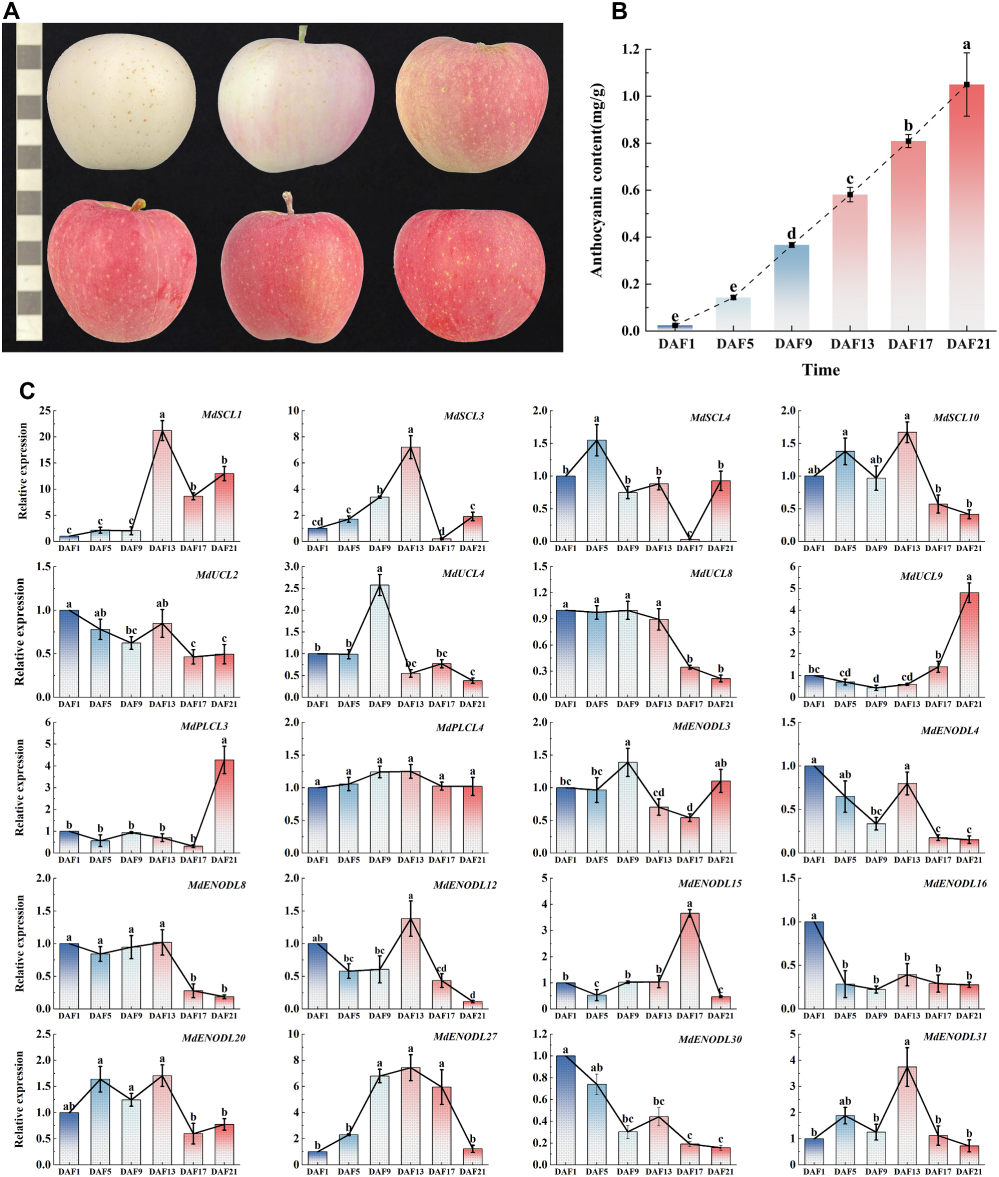
Expression levels of *MdPC* gene at different periods after apple bag removal. **A** Six different coloring periods (days) after fruit de-bagged. From left to right and top to bottom, they are DAF1, DAF5, DAF9, DAF13, DAF17, and DAF21. One small square represents one centimeter. **B** Anthocyanin content of apples at six different periods after de-bagging. Different colors represent different periods. **C** Expression levels of *MdPC gene* at different periods. Statistical analysis was performed by one-way ANOVA and Tukey’s honestly significant difference (HSD) test. The expression level of the control group that was not stressed has a value of 1. Black error lines represent the mean ± SE of three biological replicates. Different letters denote significant differences, whereas the same lowercase letters indicate no statistical difference (*P* < 0.05)

To investigate the response pattern of *MdPC* genes during different coloring stages of apple fruits, this study analyzed the expression of 20 *MdPC* gene members randomly selected from different subfamilies of *MdPC* from the period of freshly de-bagged and uncolored to the period of the full coloring of fruits (a total of 6 sampling times) (Fig. 11A). The results showed that the pigmentation and anthocyanin content of the pericarp increased gradually from DAF1 (day after fruit bag removal) to DAF21(Fig. 11B). The expression of *MdPC* genes can be categorized into four types (Fig. 11C), one is that *MdPC* genes are up-regulated in expression in all five periods, such as *MdSCL1*, *MdENODL27* and *MdPLCL4*. another is that *MdPC* genes are down-regulated in expression in all five periods (without the DAF1 period), such as the three groups of members, *MdENODL4*, *MdENODL16*, and *MdENODL30*.There is also a type of *MdPC* genes that were down-regulated in individual periods, such as *MdENODL15*, *MdENODL20*, *MdENODL31* and *MdSCL3*. The last type is the rest *MdPC* gene members that were up-regulated in individual periods. Notably, the *MdPC* members of Group IV (*MdSCL1/3*, *MdENODL27*) were all most highly expressed during the DAF13 period. The *MdPC* members of Group II (*MdENODL15*, *MdENODL31*) all had the highest expression during the period of DAF21. Most Group III *MdPC* members had low expression during the five apple coloring periods.

## Discussion

### The molecular evolution of MdPC

Extensive previous genome-wide characterization of *PC* genes in many plants, for instance, 38, 110, 62, 60, 90, 74, and 230 *PC* genes identified in *Arabidopsis thaliana* [10], tobacco [23], rice [8], maize [22], soybean [4], poplar [20], and cotton [36], respectively. However, compared to other plant species, there is still a lack of *PC* genes study in apple. A total of 59 *MdPC* genes were identified in this study (Fig. 1). Compared with the 38 *PC* gene members identified in Arabidopsis by the previous authors, the number of *PC* gene families in apple is much more than the number of PC gene families in Arabidopsis [10], suggesting that gene duplication has contributed greatly to the diversity of the number of MdPC gene families during the evolution of apple. Gene duplication can be used to expand the number of members of a gene family while at the same time enabling the MdPC gene family to obtain novel functions and continuously evolve [37].

Previous studies have shown that gene duplication includes segmental, tandem, and genomic duplication, with segmental duplication being more conducive to maintaining gene function [37, 38]. The types of *PC* gene replication in other species have also been analyzed in earlier studies. Segmental and tandem duplications played equal roles in the amplification of *OsPC* genes in rice [8], whereas in Arabidopsis 12 *AtPCs* originated from segmental duplications and only 2 *PC* genes from tandem duplications [10]. Although the apple genome has 17 chromosomes, the 59 *MdPC* genes were identified as unevenly distributed across 12 chromosomes (Fig. 2A). *MdPC* genes formed gene clusters on many chromosomes, e.g., chr2 and chr15 each had 10 *MdPC* genes, followed by chr9 and chr17 each had 9 *MdPC* genes, and it is indicated that these clusters were formed by tandem duplicated sequences formation. It is suggested that tandem duplication may be the main reason for the amplification of the apple PC family.

Collinearity analysis results show that 14 intraspecies collinearity gene pairs in apple, indicating that these collinearity genes may exercise similar functions in the *MdPC* gene family. Notably, these collinearity genes occurred mainly between chromosomes 9 and 17 in apple (Fig. 6A). The collinearity gene relationships between apple *PC* genes and *PC* genes of other species results showed that there were 8, 35 and 31 homologous *PC* gene pairs in apple/rice, apple/grape and apple/strawberry, respectively (Fig. 6B). This suggests that apple *PC* genes are more closely related to dicotyledonous plant *PC* genes, that homologous genes from common ancestral exist in different species, and that the homologous gene sequences in apple are significantly conserved during the evolutionary process.

The Ka/Ks ratio can be used to measure the historical selection of coding sequences. In this study, the Ka/Ks values of all 14 collinearity *MdPC* gene pairs were less than 1, indicating strong purifying selection on the *MdPC genes* (Fig. 7A). Similarly, calculated Ka/Ks values for *AtPC* gene members and found that *AtPC* gene members were also under purifying selection (Fig. 7B). Previously in poplar, except for the *PtSC8/PtSC17* members which were under positive selection (Ka/Ks value > 1) [20], the Ka/Ks values of the rest of the members were less than 1, suggesting that the PC proteins are relatively conserved in the evolution of plants. Codon bias analysis of *MdPC* genes showed that C3s of the MdPC family were positively correlated with CAI, CBI and Fop, whereas A3S and T3S of the MdPC family were negatively correlated with CBI and Fop (Fig. 7D). This demonstrates that the base type in the 3rd position of the synonymous codon of the *MdPC* gene influences the degree of codon usage preference.

### Different structural domains can enrich the function of MdPC

Based on the characteristics of the copper ligand residues of the MdPC proteins and the differences in the structural components of the proteins, this work classified the 59 identified *MdPC* genes into four subfamilies, MdENODLs, MdUCLs, MdSCLs, and MdPLCLs, with the largest number of genes in the MdENODL subfamily and the smallest number in the MdPLCL subfamily, which were consistent with the previous research in *Arabidopsis thaliana*, and *Populus tremula* [10, 20]. It is hypothesized that due to the lack of an intact copper ion binding site in the protein backbone of the MdENODL subfamily members, four amino acid residues in the copper ligand are partially or completely replaced by other amino acid residues, resulting in a higher number of ENODL proteins compared to other subfamily members. The PC proteins are composed of four main structural domains, including one essential plastidial cyanidin-like structural domain (PLCD) and three optional structural domains [3, 4]. Based on the structural domain composition, MdPC proteins were classified into 8 classes (Fig. 1B). While in poplar and cotton they were categorized into 6 and 10 classes, respectively [20, 36]. Compared with poplar protein classification, this work also identified type I and type VII structural domain composition type members. Compared to cotton this study did not identify members of SP-PLCD-PLCD-PLCD-PLCD-ALR and PLCD-PLCD structural domain composition type. Therefore, PC members of different species may have lost some of their structural domains during the evolutionary process, but most of the members have conserved structural domains.

Arabinogalactan proteins, stretch proteins, and proline-rich proteins are considered a large class of hydroxyproline-rich glycoproteins (HRGPs) [39]. Previous studies have shown that chimeric AGPs (Arabinogalactan proteins) contain at least one ALR (Arabinogalactan protein-like region) structural domain and one unrelated structural domain [40]. In this study, all MdPC members except type V, VI and VIII members contained an ALR structural domain (Fig. 1B), resulting in a total of 51 MdPC-AGPs members out of 59 MdPC members. The 51 MdPC-AGPs members were categorized into 34 typical AGPs (Type I and Type II members) and 17 atypical AGPs proteins (Type III, Type VI and Type VII members) based on whether they also contained SP, ALR, GAS and PCLD structural domains. AGPs are involved in the regulation of life activities such as cell division, seedling growth, pollen tube elongation, interactions between plants and microorganisms, and the process of sexual reproduction in plants [40, 41]. Thus, this evidence suggests that MdPC proteins play a pivotal role in the growth process of plants.

### Structural analysis of the *MdPC* gene and its putative function in fruit coloring

It found that the gene structure and motif of MdPC members are highly conserved among all subfamilies (Fig. 5A). Most of the members contain different numbers of introns and exons, among which the number of exons is two for both MdUCL subfamily members and MdPLCL subfamily members (Fig. 5C), meanwhile, both MdUCL subfamily members and MdPLCL subfamily members contain motif 1/2/3/4/5, which suggests that they have similar functions in the evolutionary process. The distribution of the number of exons in the MdSCL subfamily members and the MdENODL subfamily members have the same exon number distribution, but all MdSCL subfamily members contain motif 3, while most of the MdENODL subfamily members contain motif 4. Among the members of the MdSCL subfamily, both MdSCL3 and MdSCL8 consist of two motifs 3/4/5/7 and one motif 1/8, which are hypothesized to have similar functions during evolution. it’s worth noting that motif 9 is present only in members of the MdENODL subfamily, suggesting that MdENODLs are evolutionarily distinct from other subfamily members and may play specialized roles in plant development and growth.

POT proteins, as proton-coupled transporter proteins of the cell membrane, can be involved in defense responses in plants and also in hormone regulatory pathways in plants [42]. Previous studies have shown that OsUCL23 interacts with POT family proteins to regulate flavonoid metabolic pathways. For example, at OXUCL23, it leads to the upregulation of Tricin, a flavonoid metabolism pathway substance, and Quercetin, a flavonol metabolism pathway substance [34]. Poulev et al. using liquid chromatography/mass spectrometry (LC/MS) analysis compared the tricin content and some additional flavonoid compositions of rice bran samples from different color pericarp genotypes and found that the purple pericarp genotype had higher levels of tricin, as well as a wider range of flavonoid types [43], indicating that the tricin content might be involved in regulating the expression of the color of the purple pericarp of rice bran. To study the molecular mechanisms underlying the different colors of cherry fruits, multi-omics analysis of plants revealed that the transcription factors SBP, bHLH, WD40, and bZIP may regulate the accumulation of flavonoids such as hesperidin and naringenin and thus be involved in the coloration of yellow sweet cherry fruits during the second stage of the coloration process, which is from green to yellow [44]. This suggests that quercetin is also related to the coloration of cherry fruits.

In a study of *GhENODL6* regulation of yellow wilt resistance in cotton, it was found that silencing *GhENODL6* in cotton led to significantly lower expression levels of both the *PAL* (phenylalanine ammonia-lyase) gene and the *4CL* (4-coumarate-coenzyme a ligase) gene, which led to a reduction in the amount of SA mediated by the phenylalanine ammonia-lyase pathway [31]. 4-Coumarin CoA ligase (4CL) catalyzes the conversion of various types of hydroxycinnamic acids to the respective coenzyme A (CoA) esters during flavonoid metabolism [45], thus directly participating in the metabolism of phenylalanine, and thus the synthesis of flavonoids. At the same time anthocyanins are also products of secondary metabolism of flavonoids and originate from the phenylpropanoid biosynthetic pathway [46]. Some studies suggest that the *4CL* gene may also regulate apple fruit coloration [47]. In addition, the anthocyanin biosynthesis pathway is an extension of the phenylpropanoid and flavonoid pathways, beginning with PAL-catalyzed aminolysis, and higher PAL abundance and transcript levels contribute to the accumulation of anthocyanins in purple tea leaves [48]. These results indicate that *PC* genes may also affect anthocyanin synthesis by directly or indirectly regulating key genes in these flavonoid pathways, which in turn play a role in fruit coloration. Nevertheless, the specific molecular mechanisms still need further experimental verification.

In this work, Cis-acting elements in a 2000 bp sequence upstream of the transcription initiation site of the *MdPC* gene were analyzed aiming at identifying the association between the *MdPC* gene cis-acting elements and fruit coloring functions. Many *MdPC* genes contained functional elements such as I-box, GT1-motif, G-box, ABRE, as-1, GARE, ARE MYBI, etc., hypothesized that the *MdPC* gene may play a role in regulating fruit coloring function (Fig. 8). At the same time, combined with the tissue microarray data, it was found that many genes (*MdSCL3, MdSCL7 and MdENODL27*) were highly expressed in mature fruits and peels (Supplementary Fig. S1). The expression of 20 *MdPC* genes at six different coloring stages of apple after bag removal was also analyzed by the qRT-PCR experiments (Fig. 11C). *MdPC* gene expression in different periods can be classified into four types, (a) one is that *MdPC* genes are expressed up-regulated in every period (excluding the DAF1 period); (b) *MdPC* genes are expressed down-regulated in every period (excluding the DAF1 period); (c) *MdPC* genes are expressed up-regulated in a specific period (excluding the DAF1 period); (d) *MdPC* genes are expressed down-regulated in a specific period (excluding the DAF1 period). It is noteworthy that the gene expression of *MdPC* gene members of Group IV (*MdSCL1/3/4/10, MdENODL3/4/27*) was distributed among all four types. Gene expression of *MdPC* gene members in Group II *(MdPLCL3/4*, *MdUCL4/8/9*, and *MdENODL20*) is distributed across types a, c and d. The genes of Group I *MdPC* gene members (*MdENODL31* and *MdENODL15*) are distributed on type d. Group III *MdPC* gene members (*MdENODL8/12/16/30 and MdUCL2*) were expressed in distribution across types b and c (Supplementary Fig. S2). Furthermore, *MdPC* gene expression (*MdSCL1/3, MdENODL27*) in group IV was up-regulated at all five apple coloring stages, but the highest expression was observed at DAF13. In contrast, most of the *MdPC* gene members in group III showed lower expression at all five apple coloring stages. This suggests that different *MdPC* genes may have different functions in regulating fruit color formation.

Several works have deeply studied the different factors affecting fruit coloring, however, there are still several phenomena that cannot be explained by the existing results. This study, based on the expression analysis of the *MdPC* gene in different fruit coloring periods and selected some *MdPC* genes that might affect fruit color, which provides new insights for the study of its function and the molecular mechanism of apple fruit coloring, as well as for the selection and breeding of new varieties of apples with high coloring, good quality, and healthy function, to promote the sustainable development of the apple industry.

## Conclusion

In this study, a total of 59 *MdPC* genes were identified and characterized by comparative analysis at the genome-wide level, and the results of protein sequence characterization showed that 51 MdPCs had arabinogalactan structures and N-glycosylation sites, respectively, 50 MdPCs contained N-terminal signal peptides, and 42 MdPCs possessed glycosylphosphatidylinositol-anchored signals. The GO annotation results showed that *MdPC* can phenylacetone analogs biosynthesis. Chip data showed that *MdENODL9, MdSCL3* and *MdSCL9* genes were highly expressed in ripe pericarp. *PC* genes perform an essential function in the regulation of plant growth, development and abiotic stress, but whether *PC* genes are involved in the regulation of plant coloration remains to be investigated. The qRT-PCR results showed that *MdSCL1* and *MdENODL27* were up-regulated at all five coloring periods, *MdSCL3* had the highest expression at DAF13, while *MdUCL9* and *MdPLCL3* had the highest gene expression at the DAF21 period. These genes can be candidates for further functional studies. The characterization of the apple PC gene family provides a new perspective for further research on the function of *PC* genes in promoting apple coloration.

## Materials and methods

### Identification of *MdPC* family gene members

To identify the *MdPC* genes, obtained Arabidopsis acid sequences to blast from the relevant database (http://www.phytozome.net) [10]. Comparison of *MdPC* gene sequences with characteristic structural domains of phytocyanin proteins using the NCBI website (https://www.ncbi.nlm.nih.gov) [49] All predicted MdPC proteins were checked for their structural domains using the online site PROSITE (http://prosite.expasy.org/) [50] and identifying the final candidate *MdPC* genes. Utilized SignalP 5.0 (http://www.cbs.dtu.dk/services/signalP/) to predict the N-terminal signal peptide (NSP) [51] of MdPCs. Used the Big-PI Plant Predictor [52] (http://mendel.imp.ac.at/gpi/ plantserver.html) and NetNG-lyc 1.0 Server soft (http://www.cbs.dtu.dk/services/NetNGlyc/) to predict their GAS and N-glycosylation sites [53], respectively. The MdPC structural domains were mapped using the online MyDomains software (http://prosite.expasy.org/cgi-bin/prosite/mydomains/).

### Phylogenetic tree construction and collinearity analysis

Apple phytocyanin protein sequences were aligned with ClustalX2 (http://www.clustal.org/clustal2/) [54]. Using the MEGA 5 software to construct the MdPC phylogenetic tree [55]. The MdPC phylogenetic tree was constructed with NJ methods and the bootstrap value was 1000. Use the online site Itol (https://itol.embl.de) to beautify nwk files. collinearity analysis between apple and other species used TBtools (https://github.com/CJ-Chen/TBtools).

### Characterization of *MdPC* genes

The *MdPC* gene information was determined from the website (http://www.phytozome.net). Used online software ExPASy to analyze MdPC protein physical and chemical properties (https://web.expasy.org/protparam/) [56]. Using software TBtools (https://github.com/CJ-Chen/TBtools) to construct a *MdPC* chromosome location map. Subcellular localization prediction of the MdPC protein was analyzed by the online website (https://wolfpsort.hgc.jp/) [57].

### Three-dimensional structure analysis of apple phytocyanin proteins

SWISS-MODEL protein structure database (https://swissmodel.expasy.org) was used to predict the 3D structure model of MdPC protein [58], and the PDB files were downloaded and visualized in Pymol software The MdPC protein structural domains were labeled according to their characteristics in Figure.

### Construction of gene structure, motif sequence analysis

The gff file of apple genome was used for mapping the *MdPC* gene structure through the software TBtools. The motif of apple phytocyanin protein was analyzed by the online website (MEME: http://memesuite.org/tools/meme) [59] and also mapped using TBtools.

### Codon usage bias analysis and selective pressure analysis

The CDS sequence of the *MdPC* gene was utilized to calculate the *MdPC* codon usage preference by CodonW software [60]. Codon usage preferences were based on previous studies [61]. Gene pairs with collinearity were calculated for selection pressure using TBtools. i.e., analyzing the ratio between the non-synonymous mutation frequency (Ka) and the synonymous mutation frequency (Ks) of the MdPC. The Ka/Ks value was mapped using Origin 2021.

### *MdPC* gene Cis-element analysis

The *MdPC* gene upstream 2 kb cis-elements were obtained from the PLACE database (http://www.dna.affrc.go.jp/PLACE/signalscan.html) [49], including abiotic stress elements, light response and hormone elements. The resulting element data was embellished with TBtools.

### Expression analyses by microarray

To analyze the expressing patterns of *MdPC* genes, the Apple MDO database was used (http://bioinformatics.cau.edu.cn/AppleMDO), and extracted *MdPC* expression data from apple different tissues. Finally, TBtools software made an electronic expression heat map.

### MdPC protein interactions network prediction and *MdPC* gene annotation

To predict MdPC family members protein interaction prediction, the STRING website was used (https://string-db.org/) and the maximum confidence level to 0.900 [62]. The resulting TSV file is embellished with Cytoscape software while its value is set to “Pitch (BC)” for graphic rebuilding. Download the files for the gene enrichment used from the eggNOG-mapper website (eggnog-mapper.embl.de) and perform Go enrichment annotation with TBtools. and utilized OmicShare Tools (https://www.omicshare.com/tools/Home/Soft/getsoft) to map GO terminology word clouds.

### Plant materials preparation

The peel of ‘Fuji’ apple was collected as the experimental material in Jingning (105.7 ° E, 35.5° N). Starting from the time when the fruit was de-bagged, every three days, healthy and uniformly growing apples were collected and the peel was extracted, and a total of six times were taken until the peel was completely colored. Each sample was accurately weighed and quickly frozen with liquid nitrogen, and stored at -80 ^◦^c. At the same time, three biological replicates were set up for samples collected each time. Subsequent apple peel RNA extraction by the CTAB method.

### Apple peel anthocyanin content at different coloring times

Using liquid nitrogen grind 1 g of apple peel using liquid nitrogen and place in a 10 ml centrifuge tube followed by 1% hydrochloric acid-methanol solution. The supernatant was extracted in an ice box under dark conditions for 1 h, during which the supernatant was ultrasonicated 3–4 times and centrifuged at 10,000 rpm for 10 min. The apple peel samples were then filtered through a 0.2 μm PES filter and analyzed using a TU-1900 dual-beam UV-Vis spectrophotometer. The absorbance of the filtrate was measured at 600 nm and 530 nm respectively, according to an absorbance of 0.1 for 1 anthocyanin unit, using 1% HCl methanol solution as a blank control solution for zeroing and repeated three times. Expression of anthocyanin content in terms of absorbance difference between 530 nm and 600 nm/g fresh weight (U) [63]. i.e., U = (OD530 -OD600)/gFW.

### Quantitative real-time PCR evaluate

Design of upstream and downstream primers for the CDS sequence of *MdPC* gene using online primers from Bioengineering (Shanghai) Co. (Supplementary Table S2) The cDNA of the sample was obtained using the Prime Script RT kit (Perfect Real Time) (TaKaRa). The *MdPC* gene expression was quantified by real-time fluorescence quantitative PCR (Mx3005p, Stratagene, USA). SYBR Green I (TaKaRa) kit was also used and the *GADPH* gene was used as an internal reference gene. The relative expression of the *MdPC* gene was determined using 2^−ΔΔCT^method [64].

### Statistical analysis of the data

Statistical data were analyzed using Excel software, calculated and collated. Triplicate qRT-PCR quantification data were analyzed by one-way ANOVA with SPSS 22.0.*P* < 0.05.

### Electronic supplementary material

Below is the link to the electronic supplementary material.

Supplementary Material 1

Supplementary Material 2

Supplementary Material 3

## Data Availability

All data generated or analyzed during this study are included in supplementary information files.
