# Supplementary material for: Molecular evolution of Phytocyanin gene and analysis of expression at different coloring periods in apple (Malus domestica)
Source: BMC Plant Biol. 2024 May 8;24:374. doi: 10.1186/s12870-024-05069-6 (PMC11077699; doi:10.1186/s12870-024-05069-6)
Supplement: Supplementary file 1 — Supplementary Material 1 [file 12870_2024_5069_MOESM1_ESM.docx]

**
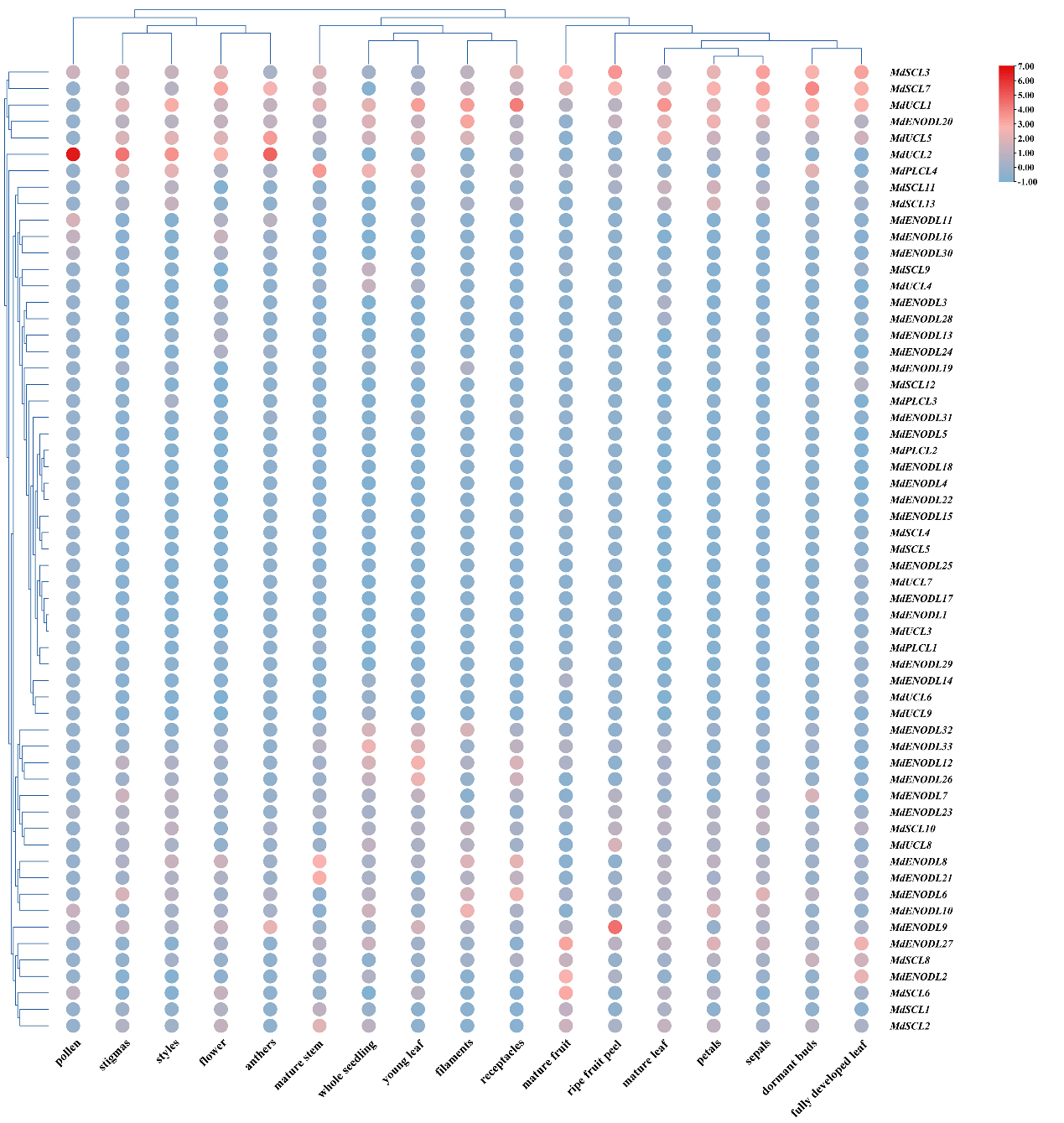
**

**Supplementary Fig. S1** Heat map of gene expression of 56 *MdPC* genes in different plant organs, Red and blue colors represent the level of gene expression, respectively


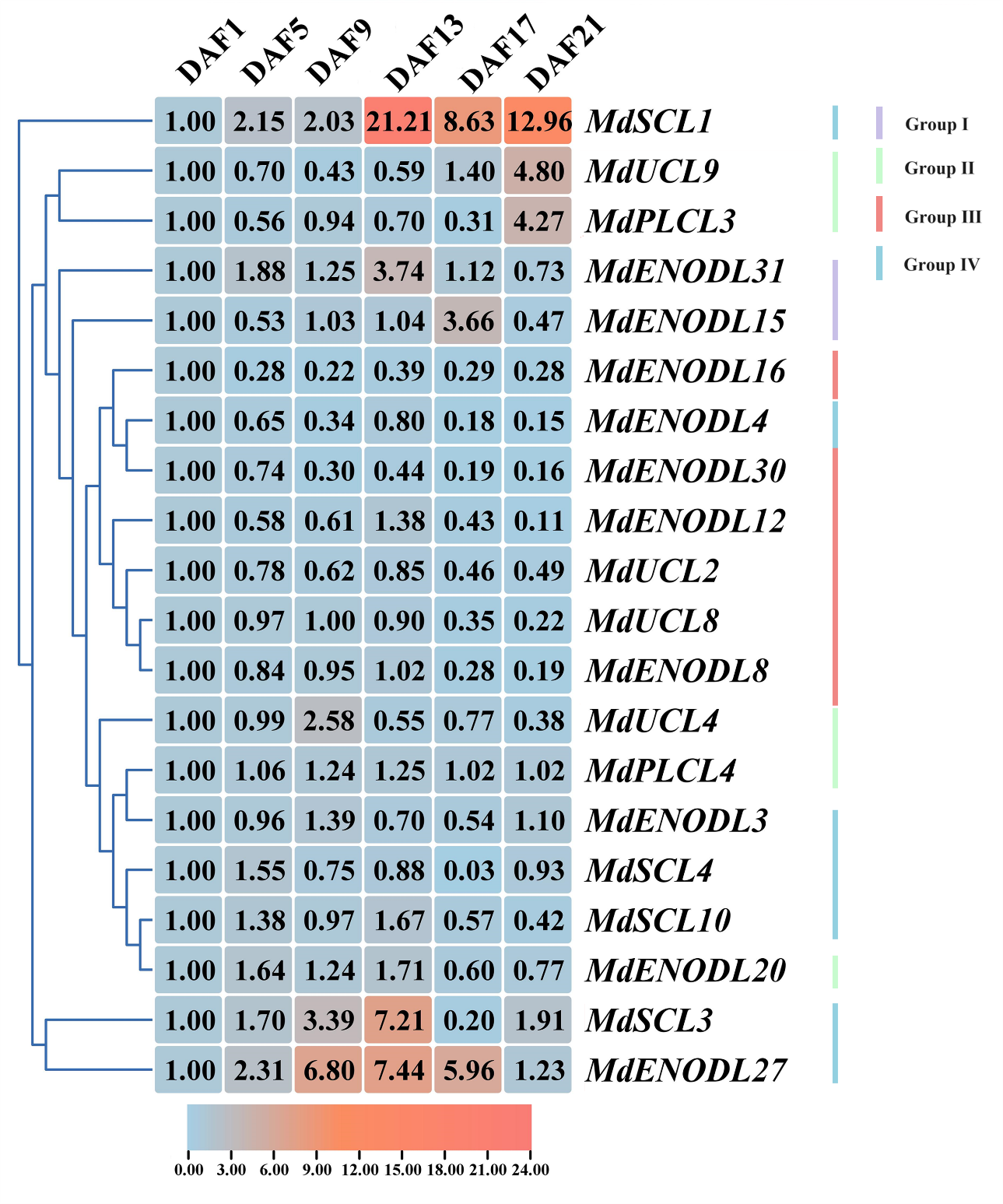


**Supplementary Fig. S2** Heat map of gene expression of 20 *MdPC* genes at different periods after apple bag removal. Orange and blue colors represent the gene expression level at different periods, respectively. The grouping of genes into different colors was based on the grouping of evolutionary trees
