# Supplementary material for: Molecular evolution of Phytocyanin gene and analysis of expression at different coloring periods in apple (Malus domestica)
Source: BMC Plant Biol. 2024 May 8;24:374. doi: 10.1186/s12870-024-05069-6 (PMC11077699; doi:10.1186/s12870-024-05069-6)
Supplement: Supplementary file 2 — Supplementary Material 2 [file 12870_2024_5069_MOESM2_ESM.docx]

**Supplementary Table S1** **Physical and chemical properties of Phytocyanin protein in apple**

| Name | Size / aa | Molecular weight / D | Isoelectric point | Formula | Instability index | Aliphatic index | Grand average of hydropathicity | Hydrophilicity | Copper-binding sites |
| --- | --- | --- | --- | --- | --- | --- | --- | --- | --- |
| *MdUCL1* | 186 | 19320.87 | 5.62 | C_852_H_1341_N_229_O_267_S_8_ | 28.55 | 88.12 | 0.156 | Hydrophilicity | H － C － H － M |
| *MdUCL2* | 187 | 20125.14 | 9.07 | C_921_H_1402_N_232_O_259_S_8_ | 36.06 | 75.61 | 0.047 | Hydrophilicity | H － C － H － M |
| *MdUCL3* | 298 | 31791.31 | 6.32 | C_1423_H_2217_N_385_O_418_S_12_ | 30.72 | 97.48 | 0.306 | Hydrophilicity | H － C － H － M |
| *MdUCL4* | 226 | 23615.82 | 5.54 | C_1057_H_1635_N_261_O_329_S_11_ | 27.99 | 87.65 | 0.254 | Hydrophilicity | H － C － H － M |
| *MdUCL5* | 183 | 18751.06 | 8.83 | C_817_H_1302_N_220_O_270_S_7_ | 58.13 | 73.01 | -0.018 | Hydropathicity | H － C － H － M |
| *MdUCL6* | 240 | 24811 | 5.45 | C_1091_H_1733_N_285_O_355_S_9_ | 75.5 | 77.71 | -0.075 | Hydropathicity | H － C － H － M |
| *MdUCL7* | 391 | 42148.12 | 6.15 | C_1870_H_2948_N_516_O_559_S_17_ | 46.9 | 92.99 | 0.066 | Hydrophilicity | H － C － H － M |
| *MdUCL8* | 174 | 17813.94 | 6.89 | C_775_H_1234_N_210_O_258_S_6_ | 39.73 | 77.41 | 0.01 | Hydrophilicity | H － C － H － M |
| *MdUCL9* | 239 | 24791.97 | 4.51 | C_1090_H_1724_N_276_O_359_S_11_ | 66.57 | 83.72 | 0.071 | Hydrophilicity | H － C － H － M |
| *MdPLCL1* | 124 | 13400.02 | 8.38 | C_590_H_915_N_161_O_186_S_5_ | 29.01 | 72.26 | -0.135 | Hydropathicity | H － C － H － M |
| *MdPLCL2* | 175 | 19490.15 | 7.69 | C_867_H_1352_N_240_O_258_S_7_ | 24.24 | 80.74 | -0.241 | Hydropathicity | H － C － H － M |
| *MdPLCL3* | 122 | 13054.9 | 8.49 | C_585_H_904_N_162_O_168_S_5_ | 0.13 | 83.93 | 0.056 | Hydrophilicity | H － C － H － M |
| *MdPLCL4* | 124 | 13136.13 | 9.55 | C_589_H_915_N_167_O_163_S_6_ | 7.7 | 81.94 | 0.142 | Hydrophilicity | H － C － H － M |
| *MdSCL1* | 172 | 18878.2 | 4.78 | C_842_H_1291_N_221_O_261_S_6_ | 35.87 | 90.12 | -0.038 | Hydropathicity | H － C － H － Q |
| *MdSCL2* | 172 | 18892.22 | 4.78 | C_843_H_1293_N_221_O_261_S_6_ | 35.87 | 90.7 | -0.04 | Hydropathicity | H － C － H － Q |
| *MdSCL3* | 345 | 34619.6 | 5.88 | C_1540_H_2359_N_401_O_490_S_9_ | 46.82 | 65.22 | 0.043 | Hydrophilicity | H － C － H － Q |
| *MdSCL4* | 195 | 20546.18 | 5.16 | C_920_H_1413_N_229_O_288_S_8_ | 42.9 | 68.15 | -0.002 | Hydropathicity | H － C － H － Q |
| *MdSCL5* | 215 | 22724.98 | 5.86 | C_1041_H_1573_N_251_O_302_S_9_ | 65.8 | 72 | -0.083 | Hydropathicity | H － C － H － Q |
| *MdSCL6* | 177 | 18545.08 | 5.02 | C_821_H_1284_N_210_O_258_S_10_ | 35.13 | 82.54 | 0.224 | Hydrophilicity | H － C － H － Q |
| *MdSCL7* | 163 | 17888.99 | 4.9 | C_798_H_1203_N_207_O_248_S_7_ | 26.98 | 80.8 | 0.043 | Hydrophilicity | H － C － H － Q |
| *MdSCL8* | 374 | 37245.26 | 5.88 | C_1643_H_2528_N_432_O_539_S_9_ | 55.44 | 61.23 | -0.045 | Hydropathicity | H － C － H － Q |
| *MdSCL9* | 222 | 23397.24 | 5.02 | C_1053_H_1601_N_265_O_327_S_6_ | 60.02 | 70.81 | -0.138 | Hydropathicity | H － C － H － Q |
| *MdSCL10* | 183 | 19152.14 | 8.47 | C_862_H_1358_N_230_O_247_S_8_ | 44.8 | 99.67 | 0.37 | Hydrophilicity | H － C － H － Q |
| *MdSCL11* | 211 | 21507.21 | 5.06 | C_961_H_1481_N_245_O_301_S_7_ | 59.22 | 77.63 | 0.218 | Hydrophilicity | H － C － H － Q |
| *MdSCL12* | 160 | 16226.1 | 5.3 | C_716_H_1104_N_184_O_234_S_6_ | 77 | 64.06 | -0.028 | Hydropathicity | H － C － H － Q |
| *MdSCL13* | 211 | 21571.26 | 5.06 | C_965_H_1481_N_245_O_302_S_7_ | 61.12 | 75.78 | 0.211 | Hydrophilicity | H － C － H － Q |
| *MdENODL1* | 169 | 18323.18 | 8.49 | C_839_H_1314_N_212_O_239_S_4_ | 39.47 | 92.84 | 0.147 | Hydrophilicity | － |
| *MdENODL2* | 305 | 32223.39 | 6.03 | C_1457_H_2221_N_375_O_436_S_8_ | 31.18 | 80 | 0.044 | Hydrophilicity | － |
| *MdENODL3* | 166 | 18141.81 | 5.06 | C_827_H_1255_N_201_O_240_S_9_ | 41.88 | 78.25 | 0.137 | Hydrophilicity | － |
| *MdENODL4* | 107 | 11874.74 | 9.12 | C_536_H_839_N_137_O_155_S_6_ | 51.46 | 73.64 | -0.105 | Hydropathicity | － |
| *MdENODL5* | 158 | 16821.26 | 5.19 | C_773_H_1162_N_186_O_221_S_7_ | 33.79 | 80.38 | 0.277 | Hydrophilicity | － |
| *MdENODL6* | 217 | 22957.61 | 4.64 | C_1017_H_1548_N_266_O_323_S_9_ | 31.35 | 66.18 | -0.148 | Hydropathicity | － |
| *MdENODL7* | 245 | 26941.1 | 4.87 | C_1204_H_1805_N_311_O_372_S_11_ | 31.48 | 75.18 | -0.206 | Hydropathicity | － |
| *MdENODL8* | 205 | 21724.19 | 4.99 | C_962_H_1468_N_256_O_305_S_7_ | 58.99 | 67.12 | -0.277 | Hydropathicity | － |
| *MdENODL9* | 367 | 37933.37 | 6.71 | C_1716_H_2581_N_441_O_522_S_6_ | 85.3 | 52.89 | -0.452 | Hydropathicity | － |
| *MdENODL10* | 196 | 21248.99 | 6.48 | C_963_H_1451_N_243_O_287_S_7_ | 49.98 | 76.12 | -0.04 | Hydropathicity | － |
| *MdENODL11* | 174 | 19393.2 | 8.32 | C_887_H_1341_N_219_O_254_S_8_ | 25.33 | 69.94 | 0.01 | Hydrophilicity | － |
| *MdENODL12* | 179 | 19145.8 | 8.5 | C_863_H_1348_N_226_O_258_S_4_ | 35.55 | 83.85 | -0.078 | Hydropathicity | － |
| *MdENODL13* | 170 | 18939.32 | 5.76 | C_852_H_1262_N_220_O_252_S_10_ | 22.71 | 62.41 | -0.068 | Hydropathicity | － |
| *MdENODL14* | 173 | 19481.43 | 5.89 | C_878_H_1345_N_227_O_251_S_12_ | 31.73 | 82.14 | -0.047 | Hydropathicity | － |
| *MdENODL15* | 129 | 14768.86 | 6.04 | C_667_H_1004_N_176_O_189_S_8_ | 27.6 | 83.72 | -0.127 | Hydropathicity | － |
| *MdENODL16* | 190 | 20889.87 | 4.75 | C_959_H_1446_N_236_O_274_S_7_ | 48.97 | 89.32 | 0.059 | Hydrophilicity | － |
| *MdENODL17* | 111 | 12611.46 | 5.97 | C_589_H_883_N_141_O_163_S_2_ | 31.36 | 114.05 | 0.37 | Hydrophilicity | － |
| *MdENODL18* | 201 | 21887.03 | 8.69 | C_991_H_1534_N_262_O_286_S_6_ | 45.84 | 85.27 | -0.008 | Hydropathicity | － |
| *MdENODL19* | 169 | 19567.43 | 8.42 | C_892_H_1347_N_237_O_245_S_8_ | 43.56 | 81.89 | -0.226 | Hydropathicity | － |
| *MdENODL20* | 281 | 30748.61 | 5.06 | C_1381_H_2086_N_348_O_423_S_13_ | 39.81 | 75.94 | -0.167 | Hydropathicity | － |
| *MdENODL21* | 214 | 22912.76 | 6.05 | C_1020_H_1573_N_271_O_316_S_7_ | 53.45 | 69.72 | -0.305 | Hydropathicity | － |
| *MdENODL22* | 166 | 17508.81 | 4.47 | C_788_H_1215_N_191_O_247_S_6_ | 32.45 | 86.45 | 0.258 | Hydrophilicity | － |
| *MdENODL23* | 198 | 21355.19 | 7.74 | C_977_H_1461_N_247_O_281_S_6_ | 37.54 | 76.41 | -0.003 | Hydropathicity | － |
| *MdENODL24* | 174 | 19155.82 | 8.4 | C_871_H_1314_N_220_O_252_S_8_ | 27.32 | 65.57 | -0.059 | Hydropathicity | － |
| *MdENODL25* | 190 | 21249.3 | 8.26 | C_964_H_1483_N_241_O_284_S_8_ | 35.72 | 78.95 | -0.12 | Hydropathicity | － |
| *MdENODL26* | 181 | 19428.01 | 7.75 | C_877_H_1354_N_228_O_263_S_4_ | 40.51 | 78.62 | -0.119 | Hydropathicity | － |
| *MdENODL27* | 307 | 32593.66 | 5.6 | C_1475_H_2251_N_377_O_448_S_5_ | 33.53 | 82.31 | 0.033 | Hydrophilicity | － |
| *MdENODL28* | 172 | 18582.17 | 5.56 | C_843_H_1295_N_209_O_252_S_6_ | 33.04 | 81.1 | 0.006 | Hydrophilicity | － |
| *MdENODL29* | 180 | 19749.72 | 9.12 | C_906_H_1389_N_235_O_251_S_5_ | 34.34 | 91.44 | 0.143 | Hydrophilicity | － |
| *MdENODL30* | 175 | 19761.33 | 5.92 | C_894_H_1309_N_229_O_257_S_12_ | 31.57 | 57.89 | -0.107 | Hydropathicity | － |
| *MdENODL31* | 172 | 19145.1 | 5.87 | C_866_H_1317_N_221_O_244_S_13_ | 39.17 | 86.22 | 0.08 | Hydrophilicity | － |
| *MdENODL32* | 169 | 18812.86 | 8.79 | C_847_H_1317_N_225_O_236_S_12_ | 37.79 | 87.57 | 0.067 | Hydrophilicity | － |
| *MdENODL33* | 176 | 19271.98 | 8.36 | C_875_H_1326_N_232_O_247_S_7_ | 35.53 | 90.23 | 0.091 | Hydrophilicity | － |

Note：－ not predicted
