# Supplementary material for: Molecular evolution of Phytocyanin gene and analysis of expression at different coloring periods in apple (Malus domestica)
Source: BMC Plant Biol. 2024 May 8;24:374. doi: 10.1186/s12870-024-05069-6 (PMC11077699; doi:10.1186/s12870-024-05069-6)
Supplement: Supplementary file 3 — Supplementary Material 3 [file 12870_2024_5069_MOESM3_ESM.docx]

**Supplementary Table S2** **qRT-PCR primers for expression analysis of *MdPC* gene in apple**

| Gene | （5′–3′） Forward primer | （5′–3′） Reverse primer |
| --- | --- | --- |
| *GADPH* | *TTCTCGTTGAGGGCTATTCCA* | *CCACAGACTTCATCGGTGACA* |
| *MdPLCL3* | GCTTAGAGGCAGTGCAATTGTTACC | ACCTTGAAGTTCCAGCCATCACC |
| *MdPLCL4* | TCCTCGCTTTGCTGCTCCAC | GTCCAACCACCTCTATCCCCAAC |
| *MdSCL1* | TGAGTCAGAAGGGATTGATCGGTTG | TTGCGTTAGCCCAGGTGGAATAG |
| *MdSCL3* | CTCCTCCTCCTCCTCCACCTTC | TGCCAACGGTCCAAGTGACAG |
| *MdSCL4* | CATCGCCGCCAACCACAATG | AGAGGCTGCTGACGGAGGAG |
| *MdSCL10* | ACTCAATCACAATCACCACCAAAGG | GGAGCAGCCGACGAAGGATG |
| *MdUCL2* | CCACATCAAGGTCGTCAACAAGC | AATGGTGGTGGCTGAGGAATGAG |
| *MdUCL4* | CTCTGGTTGGAGCACATCGGTAG | CTTGAACTGCAACTGGCGAAGTC |
| *MdUCL8* | GCGGCAGTGATGTGGTGAAAC | AGACGATGACGATGGTGATGACG |
| *MdUCL9* | CCTCGCCAGCAGAATCGGTAG | AGGGTTTGATGCAGGACTGATGG |
| *MdENODL3* | ACGAGTTGGGTTGGGCTATTCC | AGCCAAGTCTTCTTCTCCTTCAGTG |
| *MdENODL4* | AAGAAAGGGCAAAAGGTAACGGTTC | GCTTCTGTAACACTTGGACTTGGAC |
| *MdENODL8* | TTGGCAGACAGAAGCAACAGAAATG | CGCATTCGGAGGTGGAGGAG |
| *MdENODL12* | CCAAGGGACACTGCGAGAAGG | CGGAGAAGGAGCGGGAGAAATAC |
| *MdENODL15* | TGGCAGTGGTGATCGAACTTCC | ACATTGCTGGTCCAACCCATTTTC |
| *MdENODL16* | GTTTCTCTTCCATGCTTCGCTGAC | TCCATCCCAAATCATCACCCACTC |
| *MdENODL20* | GCGAGGTGTCAGTGGCAGTG | TTGGCAACCCTTGTCCGTGAG |
| *MdENODL27* | TCGCCGTTGGTGACCTTCTTG | CTTCCCTTGTGACCCTCGCTAC |
| *MdENODL30* | CGGAGGCGGAGTACAAGAAGTG | GATGAAGTAGAAGGAGCCCGAGTG |
| *MdENODL31* | GGGAGGGCGAGATGTGTATCAAC | GTGCTGGAGCGAGTTCTGGAG |
